# Genome-wide identification, expression analysis, and potential roles under low-temperature stress of bHLH gene family in *Prunus sibirica*

**DOI:** 10.3389/fpls.2023.1267107

**Published:** 2023-09-20

**Authors:** Quangang Liu, Jiaxing Wen, Shipeng Wang, Jianhua Chen, Yongqiang Sun, Qingbai Liu, Xi Li, Shengjun Dong

**Affiliations:** ^1^College of Forestry, Shenyang Agricultural University, Shenyang, China; ^2^Key Laboratory for Silviculture of Liaoning, Shenyang Agricultural University, Shenyang, China

**Keywords:** *Prunus sibirica*, basic helix-loop-helix, genome-wide analysis, low temperature stress, expression pattern, transient transformation

## Abstract

The basic helix-loop-helix (bHLH) family is one of the most well-known transcription factor families in plants, and it regulates growth, development, and abiotic stress responses. However, systematic analyses of the bHLH gene family in *Prunus sibirica* have not been reported to date. In this study, 104 *PsbHLHs* were identified and classified into 23 subfamilies that were unevenly distributed on eight chromosomes. Nineteen pairs of segmental replication genes and ten pairs of tandem replication genes were identified, and all duplicated gene pairs were under purifying selection. *PsbHLHs* of the same subfamily usually share similar motif compositions and exon-intron structures. *PsbHLHs* contain multiple stress-responsive elements. *PsbHLHs* exhibit functional diversity by interacting and coordinating with other members. Twenty *PsbHLHs* showed varying degrees of expression. Eleven genes up-regulated and nine genes down-regulated in −4°C. The majority of *PsbHLHs* were highly expressed in the roots and pistils. Transient transfection experiments demonstrated that transgenic plants with overexpressed *PsbHLH42* have better cold tolerance. In conclusion, the results of this study have significant implications for future research on the involvement of bHLH genes in the development and stress responses of *Prunus sibirica*.

## 1 Introduction

The bHLH transcription factor (TF) is one of the largest gene families shared by all three eukaryotic kingdoms (Li et al., 2020) and is widely distributed in plants, animals, and fungi (An et al., 2022). bHLH transcription factors include a highly conserved bHLH domain in their sequence that includes the basic, loop, and two helix regions (Zhuang et al., 2019). The basic region is composed of 10–20 amino acids and is located at the N-terminus of the bHLH domain, which recognizes and binds to DNA (Atchley and Fitch, 1997). The His-Glu-Arg sequence that binds to the cis-element E-box (CANNTG) in the plant bHLH domain’s basic region controls the capacity of bHLH transcription factors to bind DNA (Massari and Murre, 2000; Toledo-Ortiz et al., 2003). Two amphipathic α-helices are connected by a loop to form the HLH structure. Approximately 40–50 amino acids make up the HLH domain, which is found at the C-terminus of the bHLH domain. (Wu et al., 2021). Additionally, the HLH loop has a variable length and can form homo- or hetero-dimers, depending on its interaction with the hydrophobic amino acid region of another helix (Matsushima et al., 2004). bHLH TF was first discovered in plants in 1989 (Ludwig et al., 1989). Currently, bHLH superfamily genes have been found and studied in a variety of plant species, including *Arabidopsis thaliana* (Bailey et al., 2003), *Setaria italica* (Fan et al., 2021), *Prunus avium* (Sabir et al., 2022), and *Malus domestica* (Li et al., 2022). Based on conserved domains, phylogenetic relationships, and sequence homology, plant bHLH TFs are usually classified into 15–26 subfamilies (Buck and Atchley, 2003; Pires and Dolan, 2010), or up to 35 subfamilies if they contain atypical bHLH proteins (Carretero-Paulet et al., 2010).

The bHLH family has a large number of members and performs diverse and complex regulatory functions. It participates in a wide range of biological processes, including light signal regulation (Wang et al., 2020) and hormone signal transduction (Huang et al., 2022). bHLH TFs play important roles in different stages of plant growth and development, especially floral organ growth, anther development, and floral transition. Peach *MYB10.1* is overexpression in tobacco plants causes changes in anther filament and pistil length, and developmental defects in male and female gametophytes by repressing *NtMYB305* during flower development (Rahim et al., 2019). The *SlbHLH22* gene can promote the early flowering of tomatoes (Waseem et al., 2019), whereas *SmbHLH13* delays flowering by downregulating key flowering genes (Chen et al., 2023). *FcbHLHs* are specifically expressed in female flower tissues (Song et al., 2021). This indicates that bHLH is largely involved in floral organ development in plants. Some bHLH TFs may be activated in response to various stresses and bind to the promoters of downstream genes involved in various signaling cascades in order to control plant stress tolerance (Mao et al., 2017), such as responding to high salt, drought, and oxidative stress (Sun et al., 2018). An increasing number of bHLH TFs families have been identified with respect to their functions under low-temperature stress in plants. For example, *PubHLH1* in *Pyrus ussuriensis* activates the transcription and expression of stress response genes to significantly enhance the resistance of transgenic plants to low temperatures and freezing stress (Jin et al., 2016). *PtrbHLH* improve*s Poncirus trifoliata’s* resistance to low temperatures (Geng et al., 2019). The most well-known low-temperature response route in plants is ICE-CBF-COR (Dong et al., 2022). *MdCIbHLH1* enhances the cold tolerance of *Malus pumila* through the C-repeat-binding factor (CBF) pathway (Gao et al., 2019). The MYC-type bHLH transcription factor *ICE* is recognized as a core positive regulator of cold stress response. The expression level of *CsICE1* in *Camellia sinensis* significantly increased after low-temperature induction (Wang et al., 2012), *ICE1* and its homologous gene *ICE2* significantly enhanced the low-temperature resistance of *A. thaliana* (Xu et al., 2014).

*P. sibirica*, a deciduous shrub of Rosaceae, is native to Mongolia, eastern Siberia, and northern and northeastern China (Ma et al., 2020a). *P. sibirica* has excellent characteristics and significant ecological value that prevent wind damage, promote sand fixation, and soil and water conservation (Yin et al., 2019). In addition, *P. sibirica* has uses in food, medicine, and timber, and is an important ecological and economic tree species unique to Asia. Frost damage during early spring in northern China can easily disrupt the flowering, pollination, and fruiting of *P. sibirica*. This causes serious loss of flowers and fruits, adversely affecting the ornamental value, fruit yield, and quality of *P. sibirica* (Ma et al., 2020a). To prevent the reduction in yield and quality of *P. sibirica* due to cold damage during the flowering period, detecting cold-resistance genes is considered an important strategy for improving its resistance in *P. sibirica* breeding.

Over the past few decades, bHLH genes have undergone extensive functional analysis and genome-wide identification. However, there hasn’t been any reported functional study on the role of bHLH TFs in *P. sibirica*’s ability to withstand low temperatures. In the present study, we conducted a comprehensive genome-wide analysis of bHLH TFs in *P. sibirica*. The expression patterns under low-temperature stress and the tissue-specific expression of *PsbHLHs* were estimated using Quantitative Real-time PCR (qRT-PCR). Additionally, the gene function of *PsbHLH42* in cold tolerance was verified via transient transformation. The results of this study will help predict the function of PsbHLH members and provide a theoretical basis and technical support for the realization of *P. sibirica’s* stable yield, high-quality cultivation, and provide gene resources for future improvement of the germplasm through genetic engineering.

## 2 Results

### 2.1 Identification of the bHLH gene family members in *P. sibirica*

In total, 104 sequences with conserved bHLH domains were identified as members of the *P. sibirica* bHLH family. The physical and chemical properties of the PsbHLHs are shown in **Table 1**. The lengths of the PsbHLHs range from 91–1159 amino acids (aa), and most of these proteins (74%) had lengths of 200–500 aa. The total number of atoms ranged from 1444 (PsbHLH16) to 17840 (PsbHLH18). PsbHLH18 had the largest molecular weight (Mw) of 128.17 kDa, while in other PsbHLHs it ranged from 10.27 kDa (PsbHLH16) to 85.93 kDa (PsbHLH101). The isoelectric point (pI) ranged from 4.6 (PsbHLH22) to 10.31 (PsbHLH32), with the average pI being 6.77. Of these, 66 PsbHLHs had pI <7, indicating acidity, while 38 PsbHLHs had pI >7, indicating alkalinity. The aliphatic index (AI) ranged from 52.34 (PsbHLH50) to 98.96 (PsbHLH80). The instability index (II) ranged from 21.81in PsbHLH16 to 82.05 in PsbHLH5. A total of 101 PsbHLHs greater than 40 were stable proteins, while three were less than 40 and belonged to unstable proteins. The predicted grand average of hydropathicity (GRAVY) values were all negative and ranged between −0.972 (PsbHLH88) and −0.271 (PsbHLH83), representing that all proteins have hydrophilic properties. Subcellular localization assays revealed that 102 PsbHLHs (98.08%) were located in the nucleus; however, PsbHLH47 was located in the chloroplast, and PsbHLH71 was found in the mitochondria.

**Table 1:** Information on physicochemical properties and prediction of subcellular localization.

Gene Name	Gene ID	aa	Total number of atoms	MW (kDa)	pI	II	AI	GRAVY	Subcellular location prediction
*PsbHLH1*	PaF106G0100000075	407	6311	45281.05	5.2	58.55	74.52	−0.513	Nuclear
*PsbHLH2*	PaF106G0100000386	294	4824	33943.63	9.72	46.64	94.22	−0.297	Nuclear
*PsbHLH3*	PaF106G0100000456	111	1759	12737.3	4.92	60.35	55.41	−0.785	Nuclear
*PsbHLH4*	PaF106G0100000860	215	3467	24520.99	8.39	59.96	84.33	−0.524	Nuclear
*PsbHLH5*	PaF106G0100001763	105	1711	12006.93	10.09	82.05	89.05	−0.513	Nuclear
*PsbHLH6*	PaF106G0100002024	567	8395	60686.51	7.06	54.73	60.81	−0.666	Nuclear
*PsbHLH7*	PaF106G0100002662	206	3149	22412.42	5.73	52.2	82.48	−0.496	Nuclear
*PsbHLH8*	PaF106G0100003051	491	7632	54365.67	6.49	57.7	83.79	−0.474	Nuclear
*PsbHLH9*	PaF106G0100003177	287	4293	31102.58	6.25	59.03	59.51	−0.686	Nuclear
*PsbHLH10*	PaF106G0100003498	336	5107	36899.72	5.83	53.6	66.49	−0.803	Nuclear
*PsbHLH11*	PaF106G0100003539	387	5737	41420.98	5.86	58.33	62.48	−0.731	Nuclear
*PsbHLH12*	PaF106G0100003631	360	5640	40461.5	6.25	69.17	75.11	−0.717	Nuclear
*PsbHLH13*	PaF106G0100003722	230	3571	25856.9	5.84	39.12	75.43	−0.659	Nuclear
*PsbHLH14*	PaF106G0100003755	282	4439	31746	7.27	52.57	77.84	−0.516	Nuclear
*PsbHLH15*	PaF106G0100004903	433	6416	46677.67	5.62	59.61	61.25	−0.648	Nuclear
*PsbHLH16*	PaF106G0100004961	91	1444	10272.65	8.08	21.81	84.73	−0.389	Nuclear
*PsbHLH17*	PaF106G0100004962	388	5900	42978.47	5.27	53.04	64.1	−0.698	Nuclear
*PsbHLH18*	PaF106G0100005054	1159	17840	128177.32	6.13	54.42	76.99	−0.491	Nuclear
*PsbHLH19*	PaF106G0100005067	244	3747	26927.56	9.41	55.09	66.8	−0.504	Nuclear
*PsbHLH20*	PaF106G0100006041	229	3641	25931.49	9.75	76.73	77.07	−0.61	Nuclear
*PsbHLH21*	PaF106G0100006108	238	3638	26158.38	8.63	50.34	73.03	−0.587	Nuclear
*PsbHLH22*	PaF106G0100006225	354	5536	40196.03	4.6	59.72	71.07	−0.594	Nuclear
*PsbHLH23*	PaF106G0200006894	429	6569	48035.24	7.67	52.76	55.06	−0.856	Nuclear
*PsbHLH24*	PaF106G0200008061	484	7462	53733.92	5.52	48.5	77.33	−0.617	Nuclear
*PsbHLH25*	PaF106G0200008065	110	1760	12373.22	9.23	44.1	86	−0.615	Nuclear
*PsbHLH26*	PaF106G0200008550	489	7263	52911.85	8.38	55.54	53.7	−0.548	Nuclear
*PsbHLH27*	PaF106G0200008992	649	10149	72832.39	5.14	45.79	83.37	−0.447	Nuclear
*PsbHLH28*	PaF106G0200009226	644	9875	70841.82	6.28	49.2	75.7	−0.461	Nuclear
*PsbHLH29*	PaF106G0200009298	442	6799	48773.78	5.6	39.31	79.41	−0.523	Nuclear
*PsbHLH30*	PaF106G0200009336	414	6470	46592.42	5.7	70.25	72.34	−0.686	Nuclear
*PsbHLH31*	PaF106G0200009998	276	4250	30389.9	8.72	43.53	77.54	−0.29	Nuclear
*PsbHLH32*	PaF106G0200010400	176	2836	20283.23	10.31	62.81	61.59	−0.93	Nuclear
*PsbHLH33*	PaF106G0200010401	209	3251	22967.22	9.45	58.35	92.34	−0.314	Nuclear
*PsbHLH34*	PaF106G0200010402	126	2072	14601.9	9.87	64.59	81.98	−0.888	Nuclear
*PsbHLH35*	PaF106G0200010640	389	5903	42301.5	4.96	69.69	75.99	−0.429	Nuclear
*PsbHLH36*	PaF106G0300013995	442	6727	48826.23	6.44	52.39	66.2	−0.67	Nuclear
*PsbHLH37*	PaF106G0300013041	187	3056	21752.7	8.52	63.56	83.9	−0.688	Nuclear
*PsbHLH38*	PaF106G0300012720	290	4473	32491	6.31	42.16	58.52	−0.822	Nuclear
*PsbHLH39*	PaF106G0300012457	565	8498	61955.7	7.01	57.03	54	−0.702	Nuclear
*PsbHLH40*	PaF106G0300011956	445	6834	49394.26	6.15	71.3	66.7	−0.483	Nuclear
*PsbHLH41*	PaF106G0300011676	316	4784	34732.1	5.07	53.44	66.61	−0.819	Nuclear
*PsbHLH42*	PaF106G0300010938	544	8252	59640.79	5.52	55.7	69.39	−0.558	Nuclear
*PsbHLH43*	PaF106G0400018010	238	3672	26264.76	6.91	56.15	70.04	−0.633	Nuclear
*PsbHLH44*	PaF106G0400017995	205	3261	23251.51	5.29	52.32	81.37	−0.514	Nuclear
*PsbHLH45*	PaF106G0400017992	464	6799	49696.7	5.92	66.86	58.86	−0.498	Nuclear
*PsbHLH46*	PaF106G0400017777	186	2913	20710.67	7.78	44.14	83.33	−0.458	Nuclear
*PsbHLH47*	PaF106G0400017639	265	4019	28646.25	8.59	50.27	79.17	−0.319	Chloroplast
*PsbHLH48*	PaF106G0400017111	295	4622	33240.66	4.8	66.47	74.44	−0.391	Nuclear
*PsbHLH49*	PaF106G0400016686	324	5111	36589.59	4.93	56.11	79.23	−0.335	Nuclear
*PsbHLH50*	PaF106G0400015886	453	6823	49692.83	6.19	53.02	52.34	−0.712	Nuclear
*PsbHLH51*	PaF106G0400015827	236	3639	25787.36	7.71	48.28	77.29	−0.55	Nuclear
*PsbHLH52*	PaF106G0500018903	314	4925	35018.94	8.82	57.57	83.89	−0.401	Nuclear
*PsbHLH53*	PaF106G0500019538	484	7554	54360	7.63	43.08	76.92	−0.486	Nuclear
*PsbHLH54*	PaF106G0500019697	641	10014	71752.25	5.75	51.18	83	−0.44	Nuclear
*PsbHLH55*	PaF106G0500020042	476	7481	53708.62	8.3	47.11	79.03	−0.506	Nuclear
*PsbHLH56*	PaF106G0500020043	497	7708	55682.26	6.95	47.44	72.17	−0.577	Nuclear
*PsbHLH57*	PaF106G0500020044	489	7561	54577.17	6.42	43.94	75.11	−0.475	Nuclear
*PsbHLH58*	PaF106G0500020045	483	7498	54137.5	6.33	49.31	76.25	−0.478	Nuclear
*PsbHLH59*	PaF106G0500020046	493	7687	55424.05	6.86	53.74	73.35	−0.575	Nuclear
*PsbHLH60*	PaF106G0500020177	541	8164	58834.74	5.43	46.17	76.04	−0.52	Nuclear
*PsbHLH61*	PaF106G0500020561	189	3022	21193.48	9.59	41.77	98.47	−0.338	Nuclear
*PsbHLH62*	PaF106G0500020645	279	4138	29878.08	5.77	59.06	65.77	−0.663	Nuclear
*PsbHLH63*	PaF106G0500020717	276	4275	30576.29	5.71	49.67	77.79	−0.666	Nuclear
*PsbHLH64*	PaF106G0500020849	333	4996	36462.16	8.75	62.59	53.12	−0.727	Nuclear
*PsbHLH65*	PaF106G0500020975	577	8788	63363.39	8.52	52.2	56.85	−0.854	Nuclear
*PsbHLH66*	PaF106G0500021121	282	4343	31692.12	5.7	48.46	55.32	−0.77	Nuclear
*PsbHLH67*	PaF106G0600021631	271	4073	29545.93	8.33	50.73	59.82	−0.641	Nuclear
*PsbHLH68*	PaF106G0600022730	333	5054	36400.5	4.79	44.53	79.1	−0.42	Nuclear
*PsbHLH69*	PaF106G0600023320	310	4702	34213.74	6.16	61.38	74.06	−0.79	Nuclear
*PsbHLH70*	PaF106G0600023883	326	5060	36537.24	6.2	49.06	77.79	−0.416	Nuclear
*PsbHLH71*	PaF106G0600023926	214	3381	23646.58	9.91	47.38	90.61	−0.304	Mitochondrial
*PsbHLH72*	PaF106G0600024068	360	5588	40449.35	4.75	68.99	70.42	−0.578	Nuclear
*PsbHLH73*	PaF106G0600024087	363	5640	40149.29	6.68	54.68	76.56	−0.484	Nuclear
*PsbHLH74*	PaF106G0600024088	229	3649	25634.56	8.5	46.52	93.62	−0.348	Nuclear
*PsbHLH75*	PaF106G0600024885	188	2903	21182.46	7.2	60.78	56.01	−0.797	Nuclear
*PsbHLH76*	PaF106G0600025150	729	10725	78191.09	5.71	52.61	60.56	−0.583	Nuclear
*PsbHLH77*	PaF106G0600025451	344	5329	38629.18	5.29	52.79	75.44	−0.503	Nuclear
*PsbHLH78*	PaF106G0600025625	346	5066	36558.03	5.62	54.7	72.49	−0.351	Nuclear
*PsbHLH79*	PaF106G0700028791	251	3964	28223.83	7.05	51.27	88.21	−0.532	Nuclear
*PsbHLH80*	PaF106G0700028684	268	4309	30403.82	8.96	58.7	98.96	−0.353	Nuclear
*PsbHLH81*	PaF106G0700028683	243	3907	27712.33	6.15	62.98	90.7	−0.526	Nuclear
*PsbHLH82*	PaF106G0700028681	258	4189	29717.89	6.72	63.56	92.56	−0.345	Nuclear
*PsbHLH83*	PaF106G0700028679	255	4048	28780.65	5.76	64.33	96.31	−0.271	Nuclear
*PsbHLH84*	PaF106G0700028304	351	5415	38765.65	5.92	50.08	73.16	−0.567	Nuclear
*PsbHLH85*	PaF106G0700028295	351	5430	39353.76	8.61	54.78	62.25	−0.819	Nuclear
*PsbHLH86*	PaF106G0700027549	383	5771	41895.9	5.38	70.55	69.84	−0.461	Nuclear
*PsbHLH87*	PaF106G0700027540	270	4206	30286.6	9.38	55.81	67.93	−0.566	Nuclear
*PsbHLH88*	PaF106G0700027119	326	4955	35921.62	6.02	60.97	55.4	−0.972	Nuclear
*PsbHLH89*	PaF106G0700026830	517	7792	56449.07	5.23	46.14	73.37	−0.444	Nuclear
*PsbHLH90*	PaF106G0700026668	569	8915	63739.08	8.35	70.28	78.88	−0.431	Nuclear
*PsbHLH91*	PaF106G0700026022	482	6841	50199.15	6.32	53.38	59.21	−0.528	Nuclear
*PsbHLH92*	PaF106G0800031443	441	6698	48665.44	5.55	51.16	62.2	−0.623	Nuclear
*PsbHLH93*	PaF106G0800031047	314	4676	33643.9	6.26	46.4	72.13	−0.431	Nuclear
*PsbHLH94*	PaF106G0800030874	377	5695	41450.9	5.13	56.63	68.28	−0.636	Nuclear
*PsbHLH95*	PaF106G0800030703	344	5348	38285.53	7.05	44.5	75.38	−0.422	Nuclear
*PsbHLH96*	PaF106G0800030668	338	5219	37618.05	7.09	62.91	57.66	−0.949	Nuclear
*PsbHLH97*	PaF106G0800030602	641	9857	71851.48	5.06	45.31	72.96	−0.695	Nuclear
*PsbHLH98*	PaF106G0800030601	735	11504	83181.75	5.29	52.67	80.37	−0.444	Nuclear
*PsbHLH99*	PaF106G0800030232	585	8797	63722.82	6.13	49.29	63.57	−0.741	Nuclear
*PsbHLH100*	PaF106G0800030062	500	7499	54535.34	6.88	62.98	52.36	−0.723	Nuclear
*PsbHLH101*	PaF106G0800029859	773	11931	85930.14	6.05	48.42	79.94	−0.41	Nuclear
*PsbHLH102*	PaF106G0800029751	298	4433	31730.89	6.01	48.24	77.32	−0.363	Nuclear
*PsbHLH103*	PaF106G0800029650	511	7916	57334.5	5.08	71.39	67.4	−0.704	Nuclear
*PsbHLH104*	PaF106G0800029628	324	5036	36409.93	4.73	46.34	80.06	−0.457	Nuclear

aa, Amino acids, MW, Molecular weight, pI, Isoelectric point, AI, Aliphatic index, II, Instability index, GRAVY, Grand average of hydropathicity.

### 2.2 Multiple sequence alignment and phylogenetic analysis of the PsbHLHs family

**Figure 1:**
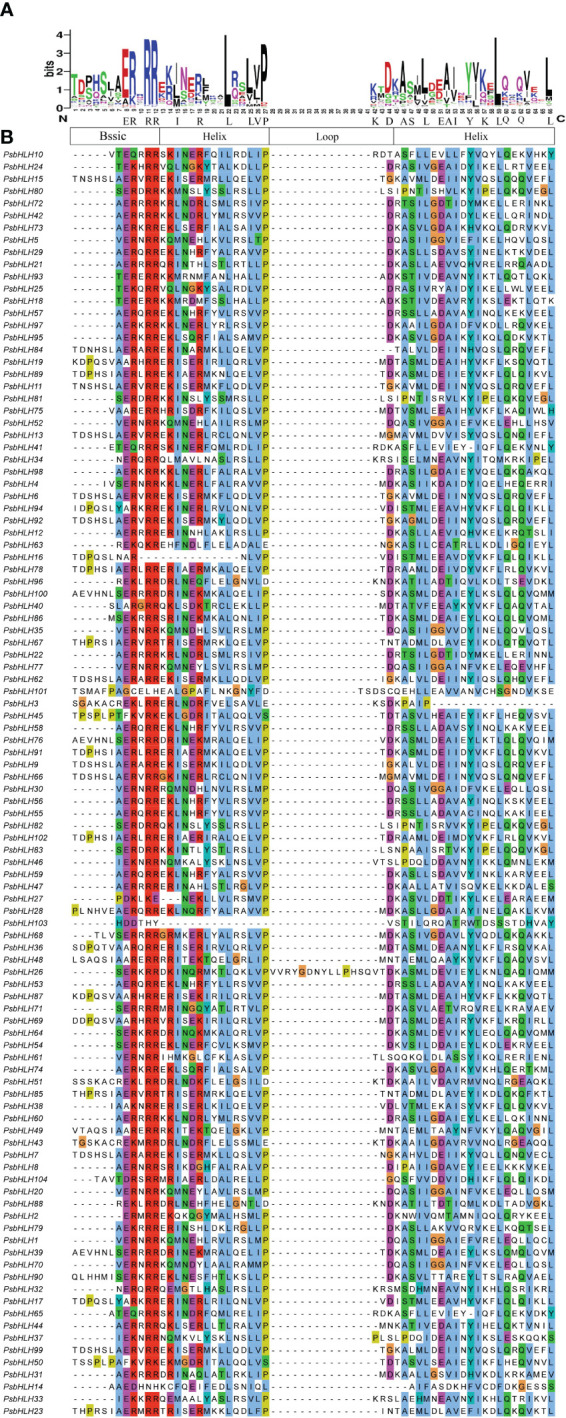
Characterization of PsbHLHs domains. **(A)** Visualization of conserved amino acids of bHLH domains. **(B)** Multiple sequence alignments of the bHLH domains using the Clustal color scheme.

Multiple sequence alignments were performed to analyze the sequence features and the presence of conserved amino acid residues in PsbHLHs. The bHLH structural domain of *P. sibirica* consisted of 66 amino acids. Visualizing the results of multiple sequence alignment allowed the identification of four conserved regions, one basic region (ERRR), two helical regions (IRLLLVP or ASLEAIYKLQQL), and a loop region (KD) (**Figure 1**). The size of the amino acid letters corresponding to each point represents the conservation of amino acids at each point in the bHLH transcription factor gene domain. The conservation of amino acid residues at 19 sites is greater than 50%. The conservation of Arg-11, Arg-12, Pro-27, Leu-22 and Leu-59 residues was greater than or equal to 90% implying the site was highly conserved. In addition, Helix1 is more conserved than Helix2.

**Figure 2:**
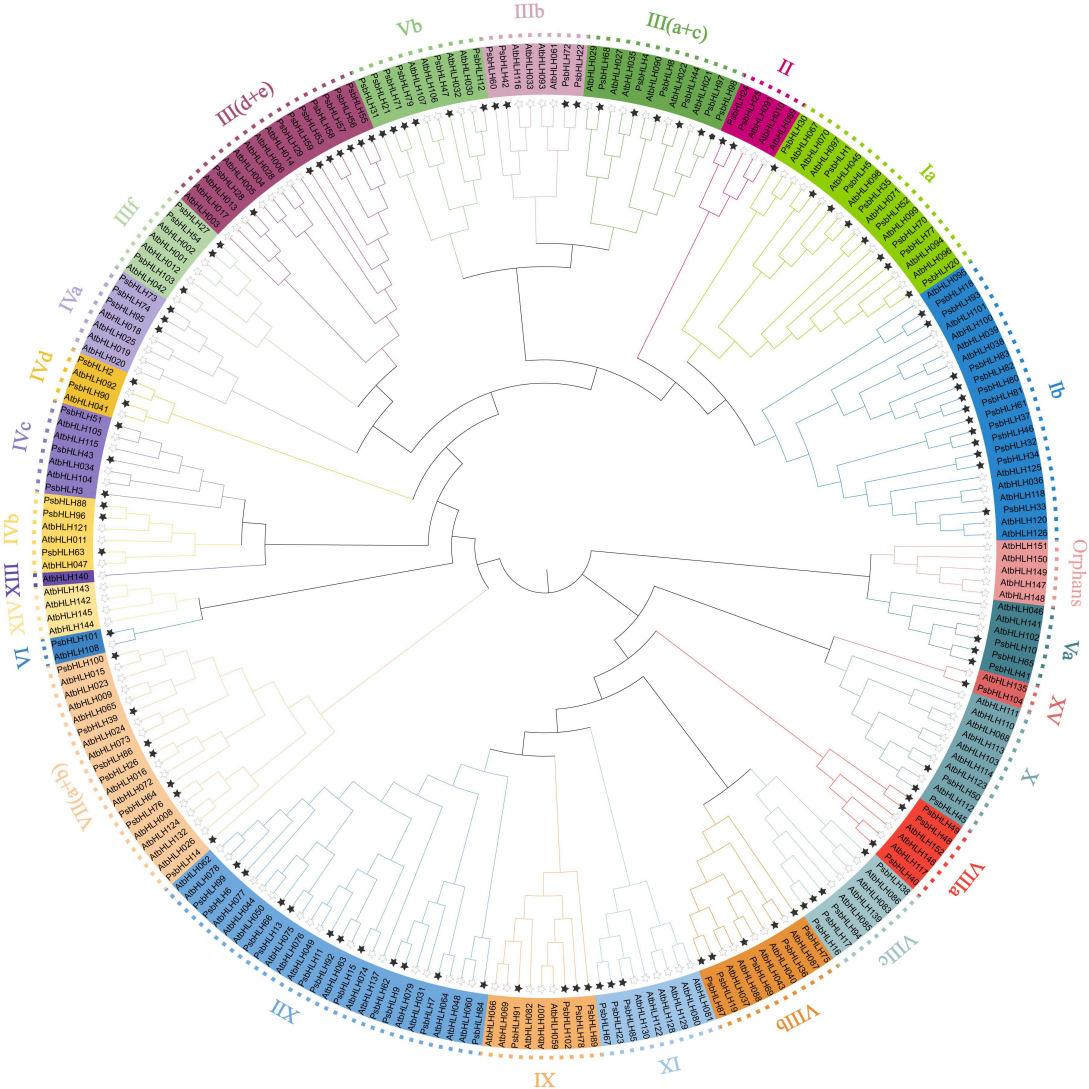
Phylogenetic analysis of the bHLH proteins of *P. sibirica* and *A. tatiana*. Different colors represent different groups of *P. sibirica* bHLH subfamilies. The black stars at the ends of branches represent *P. sibirica*, and the empty stars represent *A. thaliana*.

Using the protein sequences of the 104 PsbHLHs and 135 A*. thaliana* bHLHs, a neighbor-joining phylogenetic tree was created to examine the evolutionary relationships and classification of *PsbHLHs* members. All of the bHLH proteins were divided into 26 subfamilies based on how they were categorized in *A. thaliana*’s bHLH proteins. The nomenclature of the PsbHLH subfamilies was consistent with that of the *A. thaliana* bHLH group. Among them, there were multiple members of AtbHLHs, in the Orphans, XIV, and XIII groups, but there were none of the PsbHLHs. As shown in **Figure 2**, the Ib subfamily had the largest number of family members, containing 12 PsbHLHs; and the smallest were subfamilies VI and XV, each containing only one PsbHLH. According to the statistics of the number of AtbHLH and PsbHLH members in each subfamily, the number of bHLHs in Va, XV, VIIIa, VIIIb, VIIIc,VI, IVb, IVd, III(d+e), IIIb, and III(a+c) were the same.

### 2.3 Chromosomal localization and gene duplication of *PsbHLH*s

**Figure 3:**
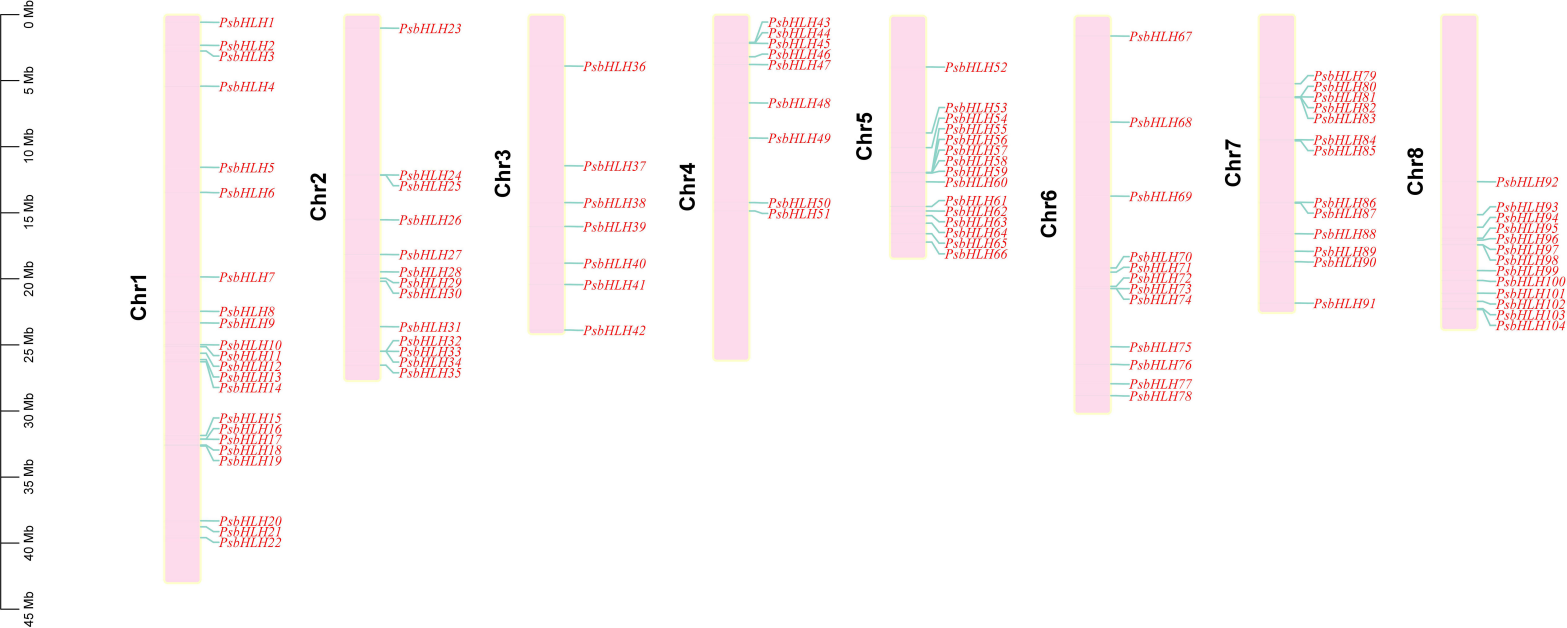
Distribution of *PsbHLHs* on 8 chromosomes. The scale bar represents megabases (Mb).

According to the genome annotation information, the 104 *PsbHLH*s were randomly distributed on eight chromosomes and numbered *PsbHLH1*–*PsbHLH104* based on their chromosomal locations, as shown in **Figure 3**. The number of *PsbHLH*s on the chromosomes ranged from seven to 22. Chromosome 1 contained the highest number of *PsbHLH*s (22). The lowest number of *PsbHLH*s was present on chromosome 3, which had only seven *PsbHLH*s. Chromosomes 2, 7, and 8 contained 13 *PsbHLHs* each, whereas chromosomes 4, 5, and 6 contained 9, 15, and 12 *PsbHLHs*, respectively (**Figure 3**). In addition, except for chromosome 3, all the chromosomes exhibited member aggregation.

**Figure 4:**
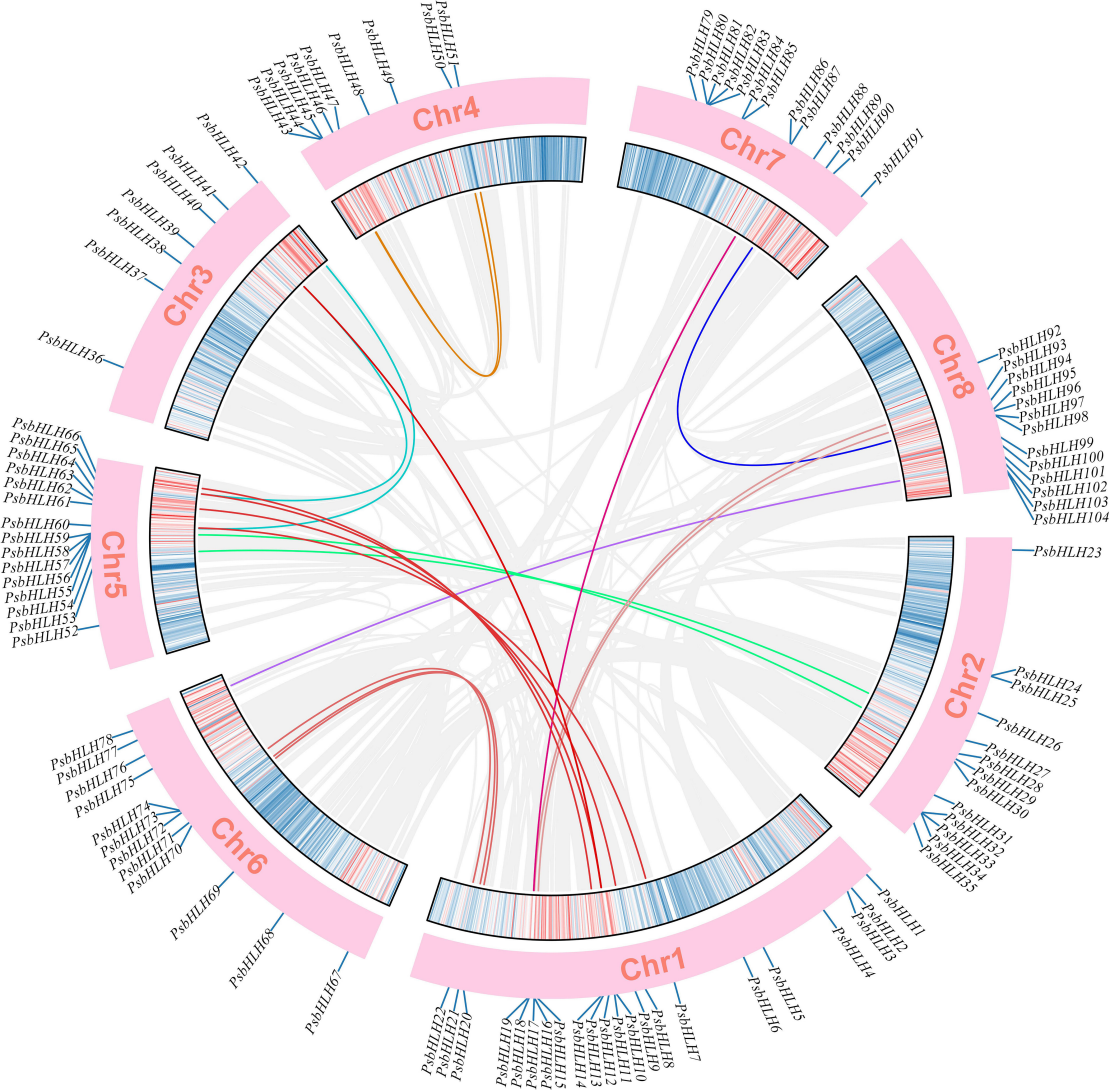
Analysis of *PsbHLH*s chromosomal distribution and duplication event. The 8 chromosomes of *P. sibirica* are represented by chr1-chr8. Duplication segments identified were connected by gray ribbons. Different colors are used to connect segmental duplicate *PsbHLH* gene pairs according to different clades. Positions are in Mb.

Gene duplication is considered the primary driving force in the evolution of genomes. Analysis of the segmental and tandem replication of the gene family plays a significant role in explaining the process of gene family expansion. In this study, we investigated the duplication events in the *PsbHLH* gene family. Gene duplication events were observed on all the chromosomes. Genes with segmental duplications were most frequently found on chromosome 1 (11 genes), followed by chromosome 5 (8 genes). In total, 48 gene duplication events representing approximately 46% (48 of 104) of the total *PsbHLHs* were found. A total of 34 genes underwent segmental duplication, which accounted for 65% of all syntenic relationships and played an essential role in the evolution of the *PsbHLHs*. These segmentally duplicated *PsbHLHs* formed 19 gene clusters that were linked to each other by colored lines, as presented in **Figure 4**. Among them, *PsbHLH10*, *PsbHLH41*, and *PsbHLH65* are duplicate gene pairs with each other. *PsbHLH18* forms two duplicate genes with *PsbHLH86* and *PsbHLH93*. Moreover, 16 (35%) *PsbHLHs*, including *PsbHLH16/17*, *PsbHLH32/33*, *PsbHLH33/34*, *PsbHLH55/56*, *PsbHLH56/57*, *PsbHLH57/58*, *PsbHLH58/59*, *PsbHLH73/74*, *PsbHLH80/81*, and *PsbHLH97/98*, were demonstrated to be tandem duplicated genes. Gene family members formed by tandem replication are closely arranged on the same chromosome and form gene clusters with related sequences and functions. Each gene pair with duplication events belonged to the same subfamily, except for *PsbHLH18* and *PsbHLH86* segmental duplication gene pairs belonging to subfamilies Ib and XIII(a+b), respectively.

The selection pressure on a gene throughout evolution can be determined by Ka/Ks ratio. The Ka/Ks values for the PsbHLH gene family were calculated (**Table 2**). Ka/Ks >1 indicates positive selection, Ka/Ks<1 indicates purified selection, and Ka/Ks=1 indicates neutral selection. All duplicated *PsbHLH* pairs with Ka/Ks < 1 might have undergone purifying selection, suggesting that the majority *PsbHLHs* evolved slowly. Therefore, the Ks value was used to calculate the divergence time of duplicated gene pairs (**Table 2**). The divergence time of these gene pairs ranged from 12.22 to 78.54 Mya. The earliest gene duplication event occurred at 78.54 Mya.

**Table 2:** The Ka/Ks ratios and estimated divergence times for duplicate pairs of *PsbHLHs*.

Duplicate gene pair		Ka	Ks	Ka_Ks	Type of duplication	Type of Selection	Divergence time (Mya)
*PsbHLH10*	*PsbHLH41*	0.38	1.67	0.23	Segmental	Purifying	55.69
*PsbHLH10*	*PsbHLH65*	0.31	1.8	0.17	Segmental	Purifying	59.93
*PsbHLH13*	*PsbHLH66*	0.33	2.13	0.15	Segmental	Purifying	71.03
*PsbHLH16*	*PsbHLH94*	0.28	0.91	0.31	Segmental	Purifying	30.21
*PsbHLH16*	*PsbHLH17*	0.49	1.69	0.29	Tandem	Purifying	56.37
*PsbHLH18*	*PsbHLH86*	0.36	2.36	0.15	Segmental	Purifying	78.54
*PsbHLH18*	*PsbHLH93*	0.35	2.28	0.15	Segmental	Purifying	76.05
*PsbHLH19*	*PsbHLH87*	0.29	2.16	0.13	Segmental	Purifying	72.05
*PsbHLH20*	*PsbHLH70*	0.51	1.78	0.29	Segmental	Purifying	59.33
*PsbHLH21*	*PsbHLH71*	0.26	1.67	0.16	Segmental	Purifying	55.8
*PsbHLH22*	*PsbHLH72*	0.33	1.41	0.23	Segmental	Purifying	47.07
*PsbHLH27*	*PsbHLH54*	0.47	–	–	Segmental	–	–
*PsbHLH29*	*PsbHLH55*	0.25	1.9	0.13	Segmental	Purifying	63.28
*PsbHLH32*	*PsbHLH33*	0.36	1.25	0.29	Tandem	Purifying	41.64
*PsbHLH33*	*PsbHLH34*	0.35	1.92	0.18	Tandem	Purifying	64.1
*PsbHLH41*	*PsbHLH65*	0.19	0.9	0.21	Segmental	Purifying	29.9
*PsbHLH42*	*PsbHLH60*	0.25	2.28	0.11	Segmental	Purifying	76.15
*PsbHLH43*	*PsbHLH51*	0.24	1.28	0.19	Segmental	Purifying	42.68
*PsbHLH45*	*PsbHLH50*	0.25	0.4	0.63	Segmental	Purifying	13.24
*PsbHLH55*	*PsbHLH56*	0.45	1.97	0.23	Tandem	Purifying	65.77
*PsbHLH56*	*PsbHLH57*	0.5	1.73	0.29	Tandem	Purifying	57.68
*PsbHLH57*	*PsbHLH58*	0.09	0.37	0.24	Tandem	Purifying	12.22
*PsbHLH58*	*PsbHLH59*	0.1	0.38	0.26	Tandem	Purifying	12.59
*PsbHLH73*	*PsbHLH74*	0.13	0.53	0.25	Tandem	Purifying	17.52
*PsbHLH78*	*PsbHLH102*	0.15	0.64	0.24	Segmental	Purifying	21.4
*PsbHLH80*	*PsbHLH81*	0.37	0.89	0.42	Tandem	Purifying	29.75
*PsbHLH88*	*PsbHLH96*	0.23	0.89	0.26	Segmental	Purifying	29.62
*PsbHLH9*	*PsbHLH62*	0.12	0.22	0.52	Segmental	Purifying	74.76
*PsbHLH97*	*PsbHLH98*	0.27	1.04	0.26	Tandem	Purifying	34.65

**Figure 5:**
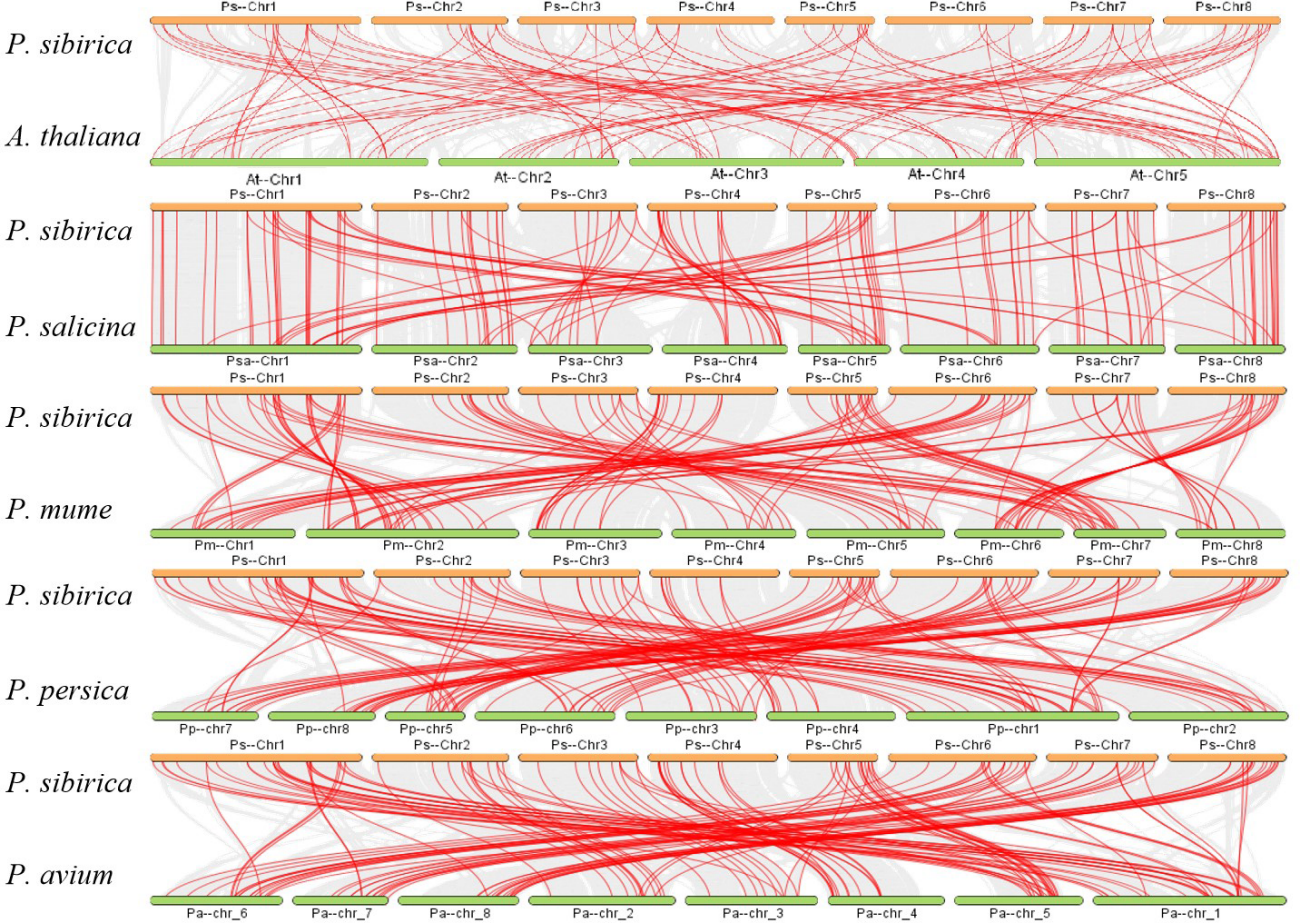
Synteny analysis of the *bHLH* genes between *P. sibirica* and five representative plant species. Gray lines are indicative of the collinear blocks within the genomes of *P. sibirica* and other plants, while red lines are indicative of the syntenic *bHLH* gene pairs. The plants named with the different prefixes “At,” “Psa,” “Pm,” “Pp,” and “Pa” represent *A. thaliana*, *P. salicin*a, *P. mume*, *P. persica*, and *P. avium*, respectively.

To learn more about the evolutionary process of the PsbHLH gene family, a syntenic map of the bHLH genes of four Rosaceae species (*Prunus salicina, P. mume, P. persica*, and *P. avium*) and the model plant *A. thalian* was constructed. Red lines represent homologous gene pairs in *P. sibirica* and other plant genomes. Between *P. sibirica* and *P. avium*, 93 orthologous gene pairs were identified, followed by *P. persica*, *P. mume*, *P. salicina*, and *A. thalian*, with 89, 88, 86, and 66 homologous gene pairs, respectively. *P. sibirica* had more homologous genes with the four plants in the Rosaceae family, which is indicative of the taxonomic relationships between the species (**Figure 5**).

### 2.4 Gene structure and conserved motif analysis

**Figure 6:**
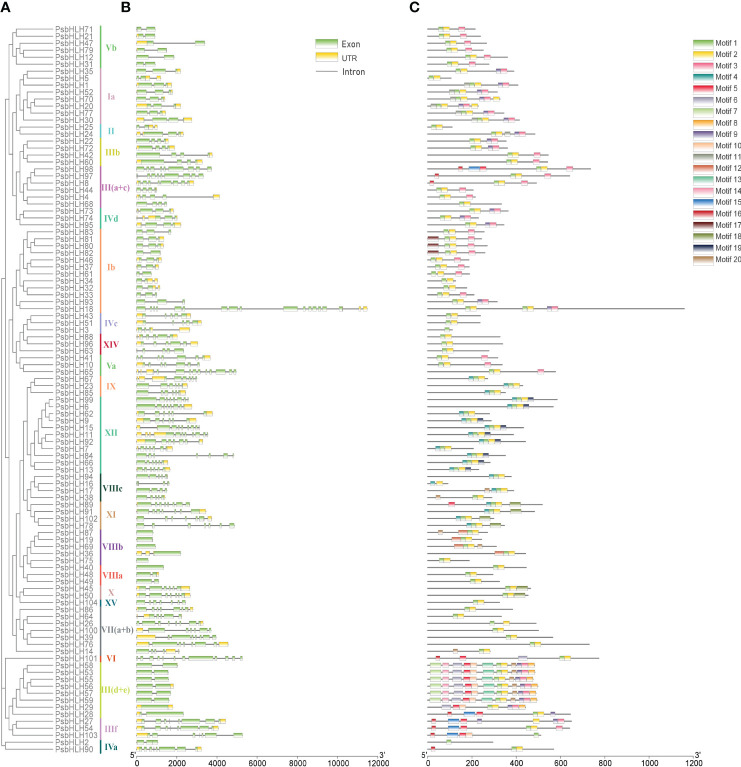
Phylogenetic relationships, conserved motifs, and gene structural analyses of the PsbHLH gene family. **(A)** Neighbor-joining phylogenetic tree of the PsbHLHs. **(B)** Gene structure of the *PsbHLHs*. The green box indicates the exons, the yellow box the untranslational regions (UTRs), and the gray line the introns. **(C)** PsbHLH proteins conserved motif distribution. Different motifs are represented by different colored boxes.

The prediction of gene structure plays an important role in understanding the evolution of gene family members. The exon-intron structures within *PsbHLHs* were investigated to determine their genetic features and structural characteristics. Different subfamilies had different intron/exon patterns, but the *PsbHLH*s sequences within the same subfamily had similar numbers of exons and introns, especially for *PsbHLHs* on homologous branches (**Figure 6**). The number of introns ranged from 0 to 17. No introns were present in *PsbHLH19*, *PsbHLH40*, *PsbHLH69*, *PsbHLH75*, or *PsbHLH89*. Exon analysis revealed that the number of exons ranged from 1 to 16. In addition, 34 *PsbHLHs* had no untranslated region (UTR). Gene structure analysis revealed differences in the structural composition of *PsbHLHs*.

Twenty motifs were identified in the PsbHLHs using Motif Elicitation (MEME). As shown in **Figure 6**, different *PsbHLHs* featured various amounts of conserved motifs, ranging from 1 to 12. Motif 1 and motif 2 constitute the conserved domain of bHLH, motif 1 contains one basic and one helical region, whereas motif2 contains one loop and another helical region (**Table S1**). Except for PsbHLH2, PsbHLH3, PsbHLH16, PsbHLH27, and PsbHLH103, almost all of the PsbHLHs contained motif1 and motif 2, and both of these conserved domains were close to one another. Interestingly, PsbHLH18 has two conserved domains (motif 1 and motif 2). PsbHLHs grouped in the same subfamily frequently shared consistent motif patterns. In addition, motifs 19, 12, 13, and 17 occurred only in subfamilies XII, VIIIb, III (d+e), and Ib, respectively.

### 2.5 Cis-acting element analysis

**Figure 7:**
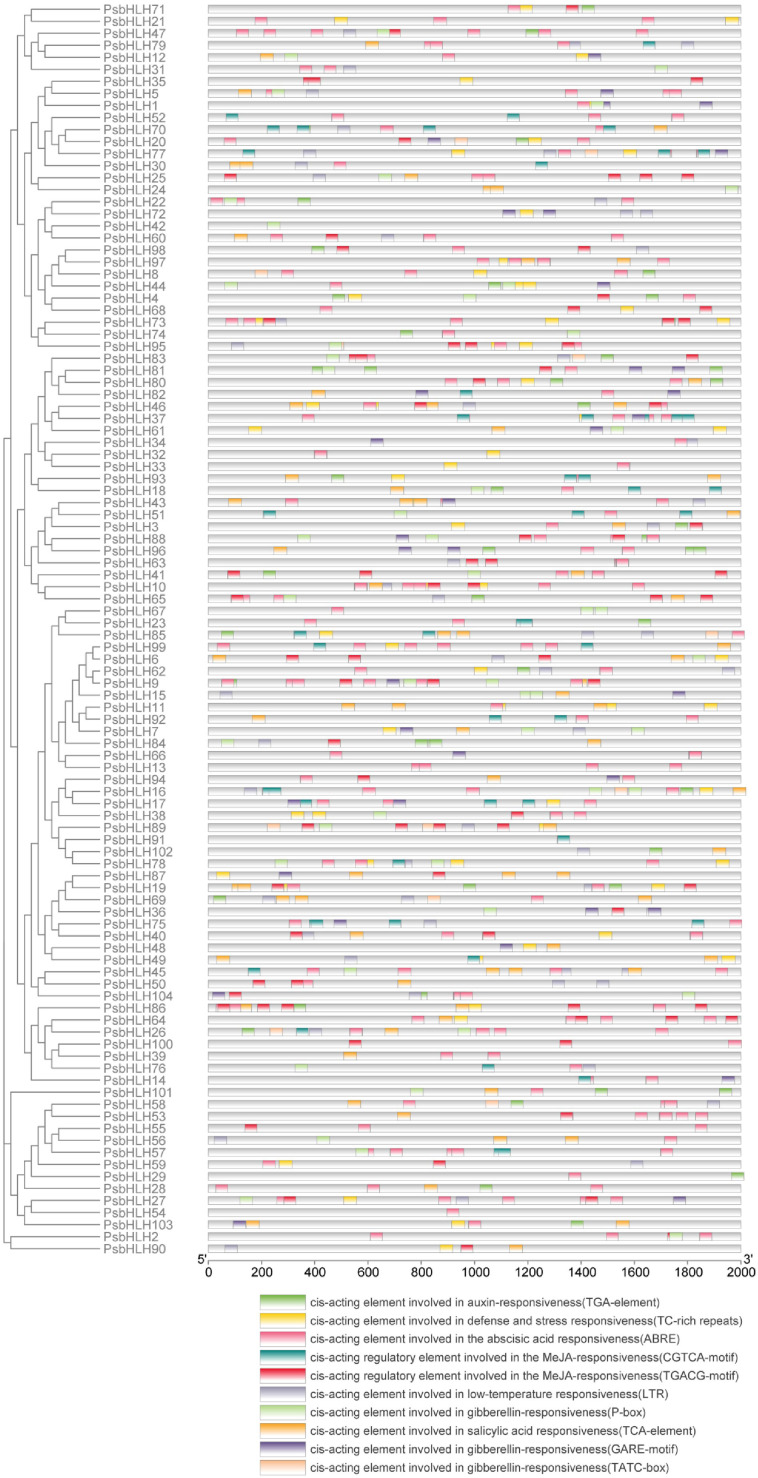
The −2000 bp sequence upstream of ATG was used as the promoter regions to identify the cis-elements. Ten cis-elements were represented in different colored blocks.

Cis-regulatory elements play an important role in regulating plant growth, development, and stress responses. To predict the regulatory pattern of *PsbHLHs*, the −2000 bp sequence upstream of ATG were analyzed in the promoter region of 104 *PsbHLHs* using PlantCARE (**Figure 7**). The discovery of numerous hormone- and stress-responsive elements suggests that *PsbHLHs* may possibly be crucial in hormone signal transduction and stress response. Cis-elements we screened were roughly divided into two categories: stress-responsive elements (TC-rich repeats and LTR) and hormone-responsive elements (ABRE, TGA-element, TCA-element, TGACG-motif, CGTCA-motif, GARE-motif, TATC-box, and P-box). Most cis-elements in the promoter regions of *PsbHLHs* were ABRE (289), followed by the TGACG motif (133) and the CGTCA motif (133). Additionally, 39 members had auxin-responsive element (TGA-element), 47 members had cis-acting element involved in defense and stress responsiveness (TC-rich repeats), 69 members had cis-acting element involved in gibberellin-responsiveness and gibberellin-responsive element (GARE-motif, TATC-boxes and P-box), 52 members had cis-acting element involved in low-temperature responsiveness (LTR), 55 members had cis-acting element that was responsive to salicylic acid (TCA-element), 89 members had cis-acting element that was responsive to the abscisic acid (ABRE), and 67 members had cis-acting regulatory element that was responsive to the MeJA (TGACG-motif and CGTCA-motif). In addition, *PsbHLH9* had the most cis-elements (22), followed by *PsbHLH86* (20).

### 2.6 Functional prediction of *PsbHLHs* based on GO annotation

**Figure 8:**
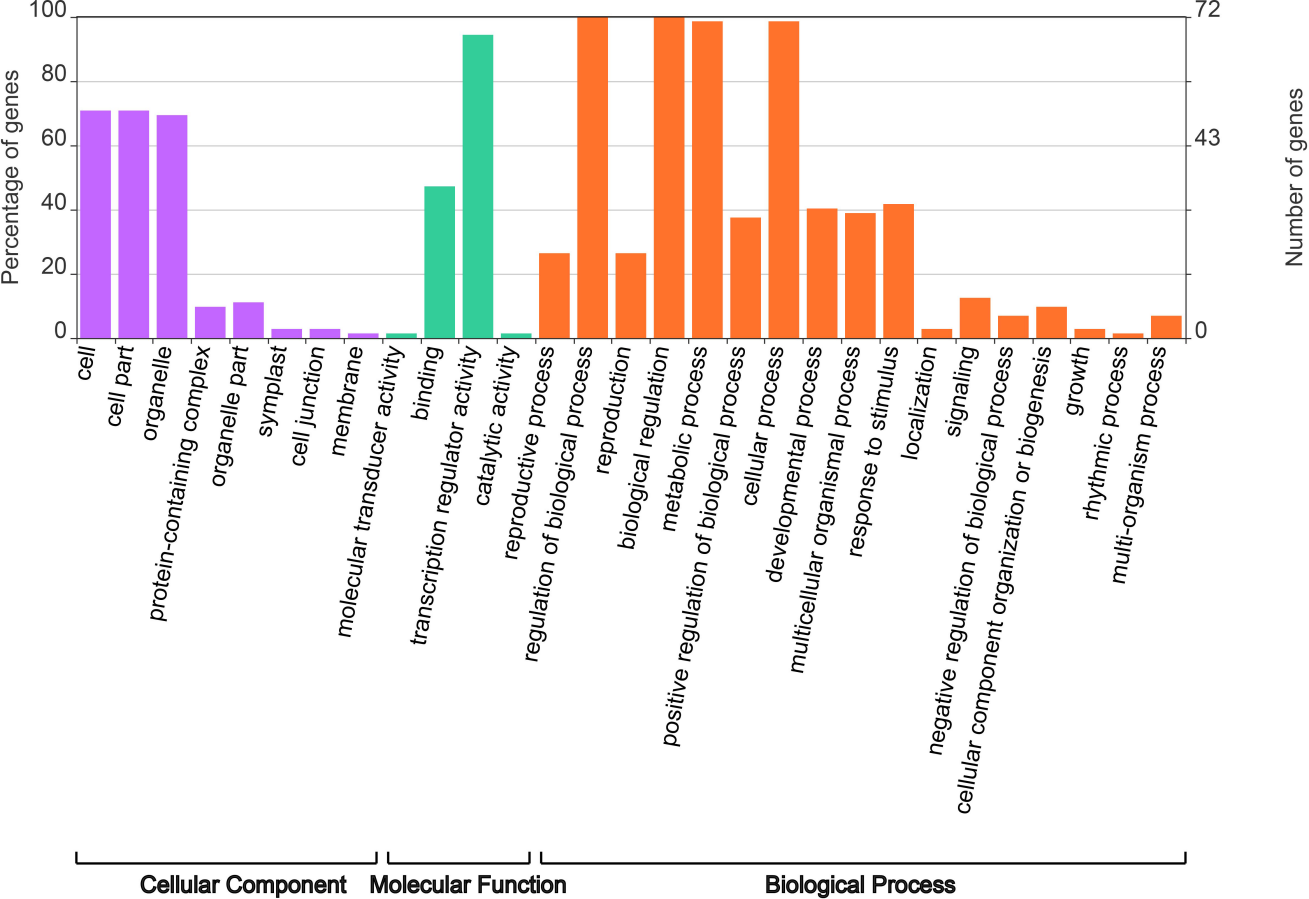
Gene ontology (GO) annotation analysis of *PsbHLHs*. GO annotation of *PsbHLHs* was performed by three categories including cellular component, molecular function and biological process.

To better understand the function of *PsbHLHs* as TFs, the EggNog website was used for gene ontology (GO) annotation analysis. Among the 104 *PsbHLHs*, 72 were annotated using GO (**Figure 8**). The results showed that 72 *PsbHLHs* were annotated to eight cellular components, 70.83% of the genes were located in cells and cell parts, 69.44% in organelles and less than 20% in other cellular components. Molecular functions include transcriptional regulator activity, binding, molecular transducer activity, and catalytic activity. *PsbHLHs* were annotated in 17 biological processes. All *PsbHLHs* were involved in the regulation of biological processes, and 71 genes (98.6%) were involved in cellular and metabolic processes. Thirty genes were involved in response to stimuli, 22 genes in the developmental processes, and 15 genes in reproductive processes. In addition, *PsbHLH16* had the most annotations (16), whereas *PsbHLH91* was annotated for 11 biological processes.

### 2.7 Expression analysis of *PsbHLH*s

**Figure 9:**
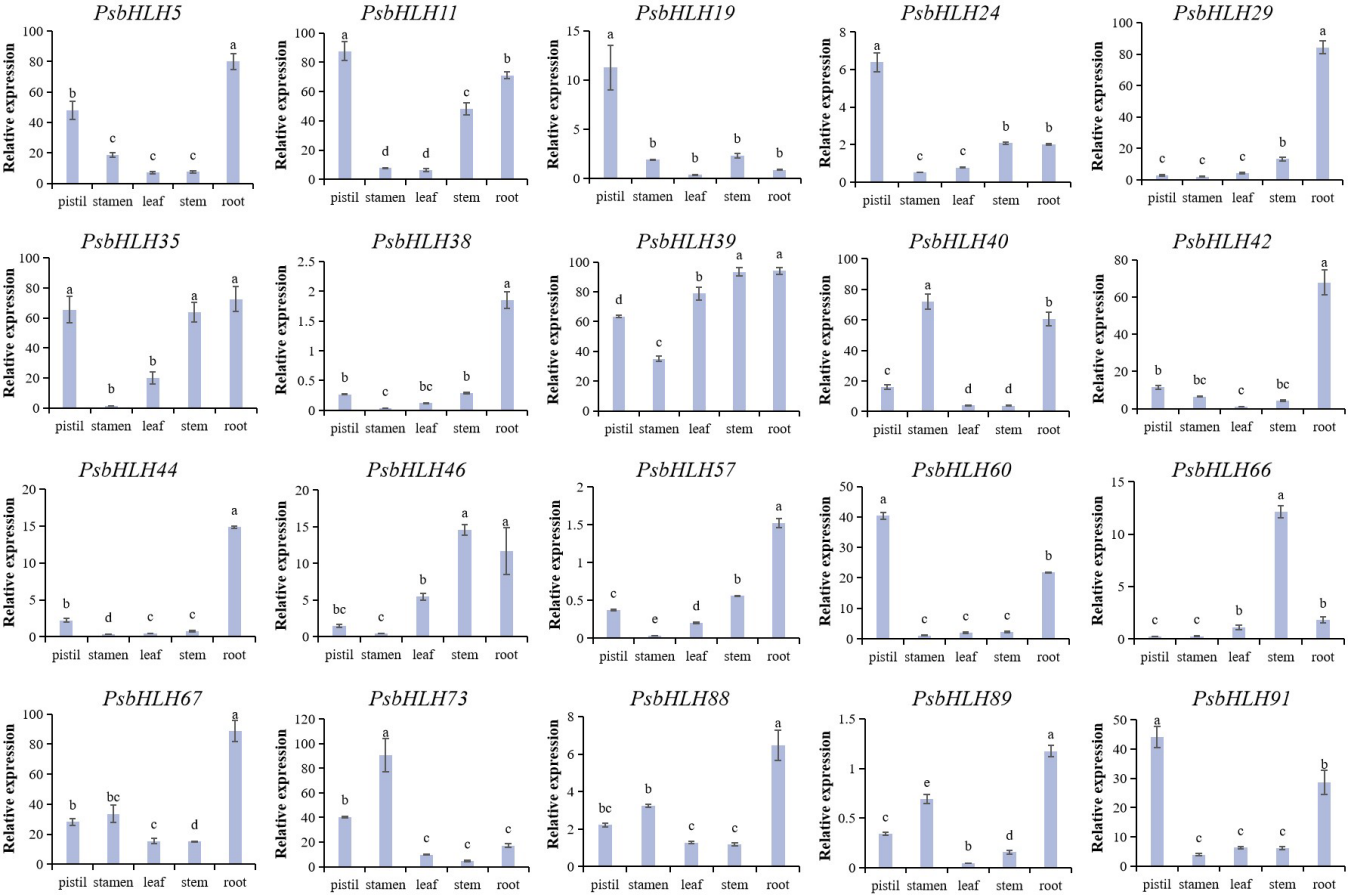
Expression patterns of the 20 *PsbHLHs* in different tissues (pistil, stamen, root, stem, and leaf) were examined by qRT-PCR. The expression level of the *18S* rRNA gene was used as the internal control. Error bars were obtained from three biological replicates and represent standard error. Significant differences achieved by Duncan’s test (*p* < 0.05) using SPSS are represented by different letters.

Twenty candidate genes were identified by integration and overall consideration of the results from the phylogenetic analysis, GO annotation, and cis-element analysis of *PsbHLHs* to further study the expression patterns and regulation processes in *P. sibirica*. These 20 genes expression levels were quantified indifferent tissues (roots, stems, leaves, pistils, and stamens) using the expression levels in petals as reference. Nine genes in pistils and 11 genes in the roots were highly expressed. Four genes were highly expressed in the stems and stamens. While, only two genes (*PsbHLH54* and *PsbHLH88*) were highly expressed in leaves. *PsbHLH39* was highly expressed in all five tissues. Five *PsbHLHs* (*PsbHLHs19*/*29*/*38*/*44*/*66*) were expressed in one tissue (**Figure 9**). *PsbHLH39* was highly expressed (>60 times) in all tissues except the stamens. The expression levels of seven *PsbHLHs* were upregulated by more than 60 times in the roots, *PsbHLH5/29/60* exceeded by 80 times; *PsbHLH11* was upregulated by 80 times in the pistil; *PsbHLH35* was upregulated by more than 60 times in the pistil and stem; and *PsbHLH73* was upregulated by 90 times in the stamen.

**Figure 10:**
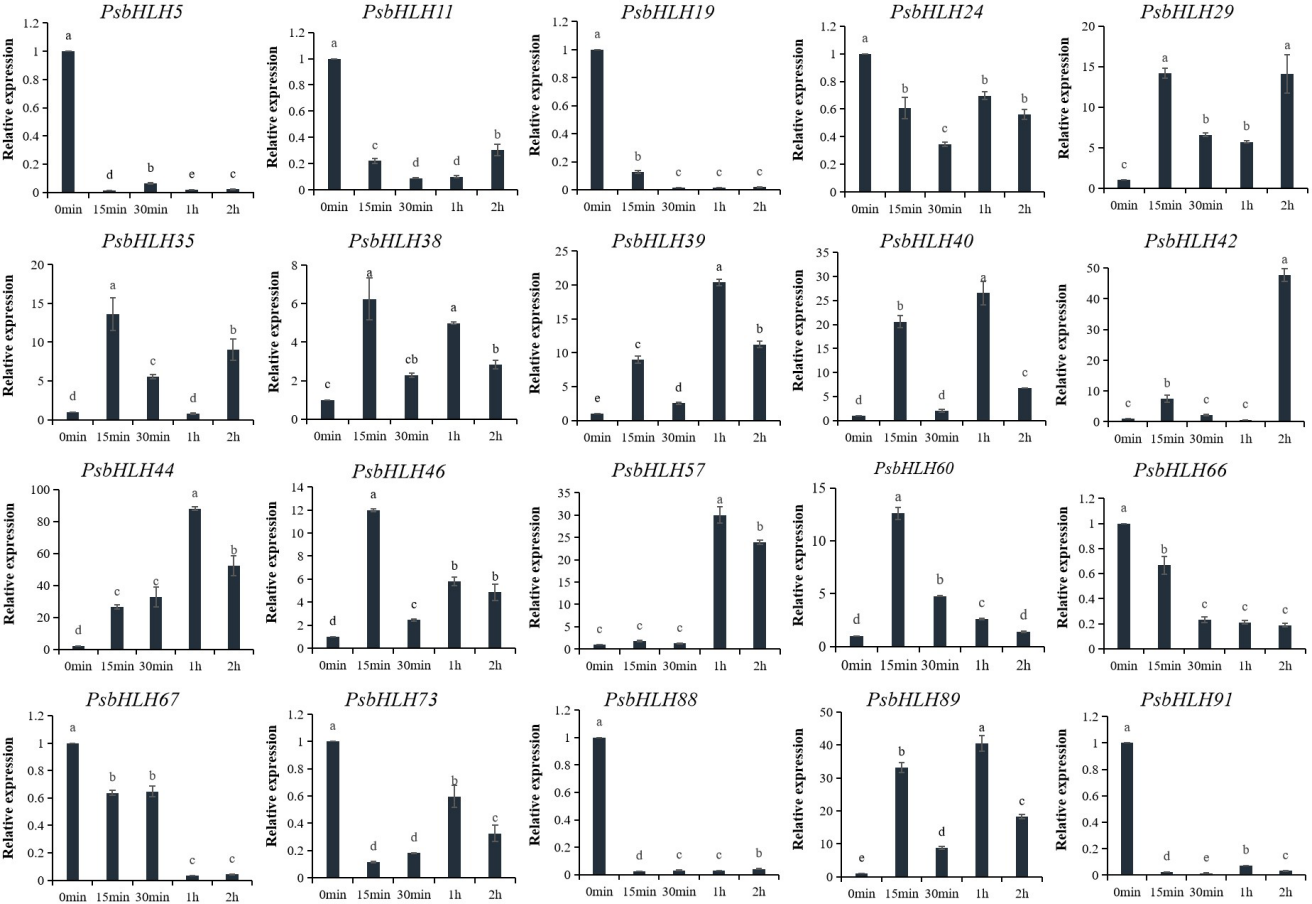
Expression patterns of 20 *PsbHLHs* in response to low-temperature stress treatment were examined by qRT-PCR. The expression level of the *18S* rRNA gene was used as the internal control. Error bars were obtained from three biological replicates and represent standard error. Significant differences achieved by Duncan’s test (*p* < 0.05) using SPSS are represented by different letters.

In order to further explore the involvement of *PsbHLHs* in low-temperature tolerance, the expression levels of 20 genes at low temperature (−4°C; 0 h, 15 min, 30 min, 1 h, and 2 h) were detected by qRT-PCR. The relative expression level (0 h) in the control pistils without treatment was normalized to 1. The expression patterns of 11 *PsbHLHs* (*PsbHLH29*/*35*/*38*/*39*/*40*/*42*/*44*/*46*/*57*/*60*/*89*) showed upregulation, whereas nine *PsbHLHs (PsbHLH5*, *11*, *19*, *24*, *66*, *67*, *73*, *88*, and *91*) displayed a downregulation. In the upregulated group, all 11 genes were highly expressed, particularly *PsbHLH44*, which displayed the highest expression level under the low-temperature treatment. Seven *PsbHLHs* (*PsbHLH29*/*35*/*38*/*39*/*40*/*42*/*89*) genes were first upregulated at 15 min, followed by slight downregulation at 30 min, and then continued to be upregulated at 1 or 2 h of treatment. In addition, the expression levels of *PsbHLH44*/*46*/*57*/*60* reached a maximum at 15 min or 1 h and then decreased with prolonged treatment time (**Figure 10**).

### 2.8 Protein interactions of PsbHLHs

**Figure 11:**
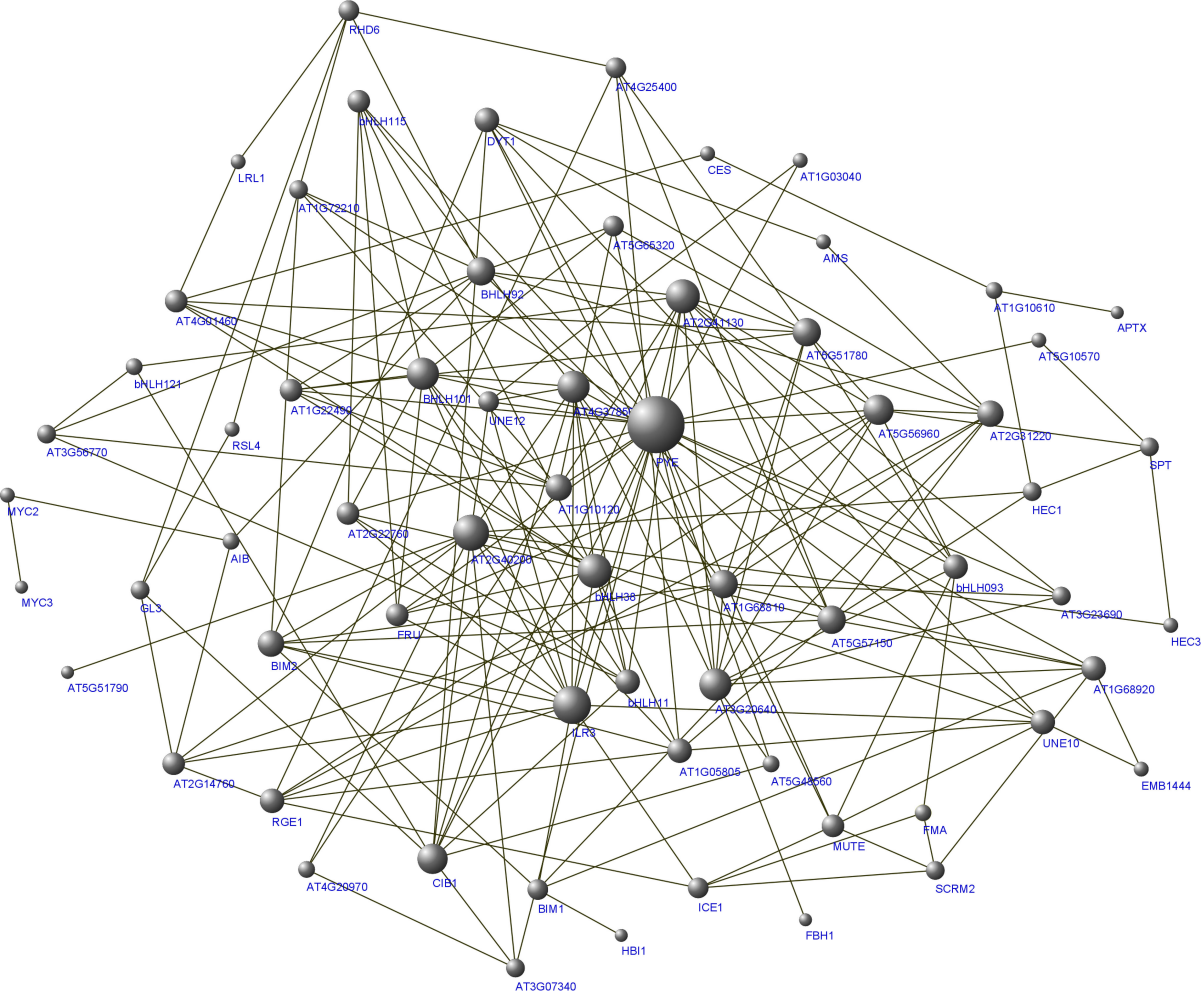
Protein interaction network for PsbHLHs according to bHLH orthologs in *A. thaliana*. The online tool STRING was used to predict the network.

Based on these syntenic relationships, orthologous AtbHLHs from 104 PsbHLHs were selected in *A. thaliana* to predict interactions. Most of the PsbHLHs were predicted to exhibit interactions (**Figure 11**). Sixty bHLHs interacted with more than one bHLH protein, with PYE (PsbHLH63) having the largest number of interacting partners (24). Detailed information on the interactions is summarized in **Table 3**. In addition, the gene function of the homolog PsbHLHs was detected based on its verified gene function in *A. thaliana*. Since *PsbHLH42* is homologous to the cold-inducible gene *ICE1*, it is very likely to take part in *P. sibirica*’s reaction to low-temperature stress.

**Table 3:** Protein–protein interaction information of PsbHLHs.

PsbHLHs	AtbHLHs	Number of protein interaction	PsbHLHs	AtbHLHs	Number of protein interaction
PsbHLH33	AT5G51790	1	PsbHLH77	AT5G65320	5
PsbHLH75	APTX	1	PsbHLH17	AT2G14760	6
PsbHLH92	HBI1	1	PsbHLH30	AT4G01460	6
PsbHLH58	MYC3	1	PsbHLH35	AT1G22490	6
PsbHLH67	FBH1	1	PsbHLH5	MUTE	6
PsbHLH101	EMB1444	2	PsbHLH68	FRU	6
PsbHLH22	AT5G10570	2	PsbHLH73	AT2G22760	6
PsbHLH66	CES	2	PsbHLH43	bHLH115	6
PsbHLH91	AT1G03040	2	PsbHLH44	DYT1	7
PsbHLH59	MYC2	2	PsbHLH72	bHLH093	7
PsbHLH69	HEC3	2	PsbHLH85	AT1G05805	7
PsbHLH89	LRL1	2	PsbHLH93	RGE1	7
PsbHLH94	RSL4	2	PsbHLH96	bHLH11	7
PsbHLH98	AMS	2	PsbHLH15	AT1G68920	7
PsbHLH1	FMA	3	PsbHLH64	UNE10	7
PsbHLH8	AT1G10610	3	PsbHLH6	AT1G10120	8
PsbHLH88	bHLH121	3	PsbHLH25	AT2G31220	8
PsbHLH95	AIB	3	PsbHLH41	BIM2	8
PsbHLH46	AT4G20970	3	PsbHLH12	AT1G68810	9
PsbHLH9	AT5G48560	3	PsbHLH2	BHLH92	9
PsbHLH20	AT1G72210	4	PsbHLH34	AT5G51780	9
PsbHLH27	GL3	4	PsbHLH4	AT5G57150	9
PsbHLH47	AT3G56770	4	PsbHLH90	AT5G56960	10
PsbHLH60	SCRM2	4	PsbHLH99	CIB1	10
PsbHLH62	AT3G23690	4	PsbHLH45	AT3G20640	11
PsbHLH84	AT3G07340	4	PsbHLH74	AT4G37850	11
PsbHLH86	SPT	4	PsbHLH81	BHLH101	11
PsbHLH87	HEC1	4	PsbHLH79	AT2G41130	12
PsbHLH102	UNE12	5	PsbHLH83	bHLH38	12
PsbHLH32	AT4G25400	5	PsbHLH31	AT2G40200	13
PsbHLH38	RHD6	5	PsbHLH51	ILR3	14
*PsbHLH42*	ICE1	5	PsbHLH63	PYE	24
PsbHLH65	BIM1	5			

### 2.9 Characterization of the role of *PsbHLH42* in *Populus ussuriensis* under low-temperature stress

**Figure 12:**
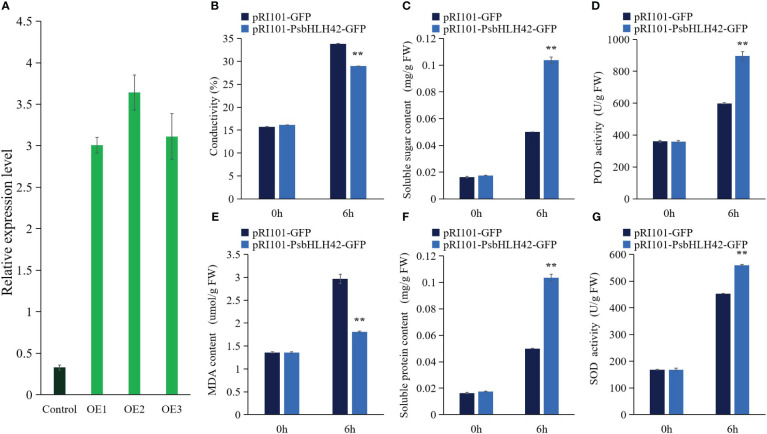
*PsbHLH42* can improve plant resistance to low temperature. Physiological indicators were determined in empty vector control and *PsbHLH42* overexpressed plants after 0 h and 6 h at 4°C low-temperature treatment. pRI101-PsbHLH42-GFP and pRI101-GFP indicate transgenic plants and empty vector transgenic plants, respectively. **(A)** Control, empty vector control plants; OE1, OE2 and OE3, three *PsbHLH42* transgenic plants. Relative expression levels of *PsbHLH42* in the transgenic and control *P. ussuriensis* plants. *18S* rRNA was used as an internal control. **(B)** Relative conductivity. **(C)** Soluble sugar content. **(D)** POD activity. **(E)** MDA content. **(F)** Soluble protein content. **(G)** SOD activity. Error bars were obtained from three biological replicates. The statistical analyses were performed using Student’s t tests (**p < 0.01).

Based on bioinformatics studies, expression pattern analysis, and homology with *ICE1*, the involvement of *PsbHLH42* in the cold response was hypothesized. To investigate the function of *PsbHLH42* in low-temperature stress, the pRI101-PsbHLH42-GFP vector was constructed and transiently transformed tissue culture seedlings of *P. ussuriensis* by the *Agrobacterium*-mediated method. An empty vector was transiently transformed into *P. ussuriensis* as the control. The qRT-PCR results demonstrated that the expression level of *PsbHLH4*2 in the transgenic plants was significantly higher than that in the control plants after transient transformation, indicating that *PsbHLH42* was successfully transferred into *P. ussuriensis* and further analysis could be carried out. The seedlings of the control and transgenic plants were subjected to 4°C stress for 6 h to evaluate the cold resistance of *PsbHLH42*. Before low-temperature treatment, the physiological signs did not alter significantly between the transgenic and control strains (**Figure 12**). After low-temperature treatment, compared with the control plants, the transgenic plants that overexpressed *PsbHLH42* had higher soluble sugar and soluble protein contents, higher Superoxide dismutase (SOD) and peroxidase (POD) activities, and lower electrical conductivity and Malondialdehyde (MDA) content.

## 3 Discussion

Basic helix-loop-helix (bHLH) TFs play a crucial roles in a variety of processes of plant growth, metabolism, and abiotic stress response (Zuo et al., 2023). However, the characterization of bHLH genes in *P. sibirica* has not been reported. In the present study, we identified 104 *PsbHLHs*. The number of bHLH TFs varied greatly among the different plants. The number of members in the *P. sibirica* bHLH gene family is smaller than that of many other plants, such as *Oryza sativa* (183) (Wei and Chen, 2018), *Triticum aestivum* (225) (Guo and Wang, 2017), *Gossypium hirsutum* (437) (Liu et al., 2019), and *Zea mays* (208) (Zhang T. et al., 2018). However, it is similar to members of the Rosaceae family, such as *P. salicina* (95) (Zhang C. et al., 2018), *P. mume* (100) (Wu et al., 2022), and *Rosa chinensis* (100) (Zhou et al., 2020). This indicates that the evolution of the bHLH gene family is highly conserved from herbaceous plants to woody plants. The fact that 98.08% of *PsbHLHs* (102) were located in the nucleus, indicates that *PsbHLHs* mainly function as TFs in the nucleus. Meanwhile, *PsbHLH47* is located in the chloroplasts, and *PsbHLH71* is located in the mitochondria. It has been speculated that these genes are mainly involved in the transcriptional regulation of photosynthesis or functional mitochondrial transcription, such as respiration, in *P. sibirica*.

Structural variation plays a crucial role in the process of gene evolution. Introns evolve with the evolution of the Genome evolution mainly represented in the gain, loss, insertion, or deletion of introns and exons (Zhao et al., 2022). The functions of genes in the identical subfamily can also be comparable or there may additionally be functional differentiation, according to the results of gene structure analysis, which revealed similarities and differences among members of the same subfamily (Zhang et al., 2022). The five genes identified without introns were all distributed in the VIIIb subfamilies, which proved that the subfamily classification was accurate. Except for PsbHLH2/3/16/27/103, all other PsbHLHs contained complete conserved bHLH domain (Motif 1 and Motif 2), suggesting that these five proteins have bHLH domains but are incomplete and belong to special bHLH family members (Carretero-Paulet et al., 2010). In addition, PsbHLH18 has two conserved domains (double motif 1 and motif 2), suggesting that it has undergone certain mutations or replication of the conserved domain during evolution, which may have improved its binding (interaction) ability with downstream genes. In addition to the bHLH domain, other conserved motifs also perform other key functions that are essential for each subfamily. Proteins in the same subfamily have similar conserved motif patterns, indicating that they may have similar functions (Eulgem et al., 2000). Some motifs are unique, with motifs 12, 19, 13, and 17 present in only one subfamily. The uniqueness and conservation of these motifs are also evidence of the evolutionary classification of the PsbHLH gene family. Interestingly, PsbHLH28 and PsbHLH98 belong to the III (d+e) and III (a+c) subfamilies but are closer to the IIIf subfamily in motif patterns. These results indicate both functional conservation and differentiation in PsbHLHs.

Gene duplication plays a crucial role in the acquisition of new genes and supplies the building blocks for the development of genetic diversity (Ma et al., 2021). Duplication analysis indicated that the PsbHLH gene family expanded through segmental (65%) and tandem (35%) duplication events, with segmental duplication being the main mechanism of amplification. The duplicated genes take new functions, whereas the original function of the other gene copy is maintained in the homologous genes (Baldini et al., 2022). Except for *PsbHLH18* (subfamily Ib) and *PsbHLH86* (subfamily VII (a+b)), all duplication events occurred within the same subfamily, suggesting that after segmental replication, new genes (*PsbHLH18* or *PsbHLH86*) may undergo functional differentiation. Ka/Ks values can be used as indicators of the selection pressure on a gene during evolution (Chen et al., 2022). Due to the degeneracy of the code, sites within codons were defined as synonymous substitutions (Ks) when they did not result in an amino acid change, and as a non-synonymous substitutions (Ka) when they did (Harouaka et al., 2016). Generally, Ka/Ks >1 indicates positive selection, Ka/Ks =1 indicates neutral selection, and Ka/Ks <1 indicates purified selection (Lin et al., 2021). Based on the Ka/Ks ratio, all PsbHLH pairs were subjected to purifying selection.

Due to segmental or tandem duplication and gene loss events, various species have variable numbers of subgroups and genes per subfamily (Sheng et al., 2022). Phylogenetic tree analysis showed that the 104 PsbHLHs could be divided into 23 subfamilies, consistent with the results for *Carthamus tinctorius* (23) (Hong et al., 2019). The lack of PsbHLHs in the three subgroups (Orphans, XIV, and XIII), when compared to *A. thaliana*, may be due to gene loss during the evolution of *P. sibirica* or natural selection leading to elimination; this reflects that the bHLH gene is highly conserved during the evolution from herbs to woody plants. There was only one member each in subclasses XV (PsbHLH104) and VI (PsbHLH101), suggesting that the members of these subclasses evolved relatively slowly, were relatively functionally conserved, and may play an important role in *P. sibirica*.

Cis-regulatory elements (CREs) in promoter regions exert a significant influence on gene functions (Hou et al., 2021). The promoter prediction results indicated that PsbHLHs contained a large number of hormone and stress response elements. *PsbHLH16* contains eight types of elements, *PsbHLH37* and *PsbHLH86* contains 10 MeJA elements, and *PsbHLH9* contains 10 ABA elements, indicating that these genes are most likely to be induced by different hormones to affect development or stress in *P. sibirica*. GO annotation revealed that the regulatory functions of *PsbHLHs* were diverse and complex. According to annotations, 69% of *PsbHLHs* were participating in the regulation of biological processes. Anthocyanins are naturally occurring plant pigments with antioxidant properties (Taghavi et al., 2022). Previous research has demonstrated that TT8 regulates anthocyanin biosynthesis (Ahn et al., 2022), and it has been speculated that the homologous gene *PsbHLH103* participates in anthocyanin synthesis in *P. sibirica*. In addition, *PsbHLH44* and *PsbHLH97* interact and are both annotated to be involved in reproductive and developmental processes; their homologs, *DYT1* (*PsbHLH44*) and *AMS* (*PsbHLH44*), are both involved in stamen development in Arabidopsis, suggesting that *PsbHLH44* and *PsbHLH97* are most likely to interact and regulate stamen development in *P. sibirica*. Similarly, *PsbHLH36* (*HEC3*) and *PsbHLH100* (*SPT*) may interact to regulate *P. sibirica* pistil development.

To a certain extent, gene expression patterns reflect their function. bHLH family members are expressed to varying degrees in different organs of *P. sibirica*. Many *PsbHLHs* are highly expressed in the roots, which is in line with the conclusions in *P. mume* (Wu et al., 2022). *PsbHLH38* is expressed in roots, and the homologous gene *RHD6* guides root hair development in Arabidopsis through processes involving auxin and ethylene, suggesting that *PsbHLH38* is also involved in root development. According to previous reports, MYC participates in the Jasmonic acid (JA) signaling pathway and directly or indirectly controls stamen development through the ABA-JA pathway (Li et al., 2023). *PsbHLH40/73* contains five ABAs and 4/6 MeJA, respectively, suggesting that they may be involved in the development of *P. sibirica* stamens. *AtHEC1* is involved in female development and fertility (Gremski et al., 2007), and its homologous gene, *PsbHLH19*, is highly expressed, and is speculated to regulate pistil development. Therefore, *PsbHLHs* play an important role in the growth and development of different organs in *P. sibirica*.

Low-temperature stress (late frost damage) severely affects the yield and quality of *P. sibirica*. To further investigate the function of *PsbHLHs* in low temperature regulation, the expression profiles of *PsbHLHs* under low temperature stress were analyzed. Twenty *PsbHLHs* exhibited different expression patterns under low-temperature treatment. For example, *MYC2* (homolog of *PsbHLH29*) is a core transcription factor of the JA signaling pathway that reduces damage to plants during abiotic stress by participating in the regulation of JA-inducible genes (Oña Chuquimarca et al., 2020; Xu et al., 2022). Studies have shown that MeJA can improve plants’ freezing tolerance by reducing membrane lipids and maintaining their stability (Ma et al., 2020b). In this study, *PsbHLH29* was highly expressed after 15 min and 2 h of cold stress. This may be the result of plasma membrane damage in *P. sibirica* during the early stages of cold induction. Initially, JA was induced to regulate membrane permeability after reducing plant damage, after that decrease expression; again, JA was expressed as the stress time increased and the degree of cell membrane damage increased. This suggests that *PsbHLH29* may regulate cold resistance in plants by regulating JA. *AtAIB* positively regulates the ABA response in Arabidopsis (Li et al., 2007). PsbHLH53(MYC3)/95(AIB) contained ABA response cis-elements, suggesting that *PsbHLH53/95* may also participate in *P. sibirica* cold response by regulating ABA. PPI analysis revealed that *PsbHLH29* (*MYC2*) interacts with *PsbHLH53/95*, suggesting that ABA and JA operate together to regulate the freezing tolerance of flowering organs of *P. sibirica*. The number and kind of cis-acting elements on the promoter may also have a bearing on how well a gene functions, with *PsbHLH44* containing three gibberellins-responsive elements that were upregulated 80-fold in response to low-temperature stress. Meanwhile, 89 contained eight MeJA-responsive elements that were also up-regulated 30-fold early (within the first 1 h), suggesting that these two genes are induced by gibberellins and MeJA to regulate the freezing resistance of *P. sibirica* flowering organs. *PsbHLH35, 38, 46*, and *60* expression reached its highest level after 15 min of cold treatment. This phenomena could have several causes, one of which is that during the initial phases of cold treatment, active regulatory factors are quickly engaged and operate on the bHLH protein to adapt to the stressful environment, which causes rapid upregulation of bHLH. To avoid an excessive amplification of stress signals, plants must maintain a balance between normal development and adaptation to cold settings when the cold treatment time is extended. At this moment, negative regulators become active and encourage the breakdown of the bHLH protein, delaying the expression of the bHLH gene (Tan et al., 2021).

Studies have demonstrated that *ICE1* and *ICE2* improve the freezing tolerance of transgenic plants (Liu et al., 2018; Ma et al., 2023). Here, *PsbHLH42* (*ICE1*) was significantly upregulated after −4°C treatment, suggesting that it may play an important role in the cold stress response of *P. sibirica*. *PsbHLH60* interacts with *PsbHLH42* (*ICE1*) and contains a low-temperature response element, whose homologous gene *SCRM2* encodes *ICE2*, suggesting that *PsbHLH42* and *PsbHLH60* may co-regulate the *P. sibirica* cold response. In addition, nine genes were downregulated after low-temperature stress, which may have been caused by the brief stress period (2 h) that was insufficient to induce gene expression. In conclusion, the above results suggest that the PsbHLH gene family may play a significant part in the cold stress response pathway.

The homolog of *ICE1* gene, *PsbHLH42*, was used to verify its function in resisting low temperatures. Transient genetic transformation assays can rapidly identify gene function (Han et al., 2023). Because the lack of mature genetic transformation and regeneration systems in *P. sibirica*, we used the ideal model tree plant *P. ussuriensis* as the genetic transformation material (Jansson and Douglas, 2007; Movahedi et al., 2014). In this experiment, the *PsbHLH42* transgenic strain showed reduced membrane damage, increased osmoregulatory substance content, and increased SOD and POD activities after low-temperature treatment compared to the control. Overexpression of the *ICE1* homologous gene *PsbHLH42* improved the low-temperature tolerance of *P. ussuriensis*. In addition, *PsbHLH42* interacts with PsbHLH5, and *MUTE* (*PsbHLH5*) participates in plant stomatal development (Lampard and Bergmann, 2007); therefore, it is speculated that they may be involved in the low-temperature response by regulating stomata. To further verify the function of this gene, stable genetic transformation experiments will be conducted in the future.

## 4 Materials and methods

### 4.1 *PsbHLHs* identification and analysis of physicochemical properties

Sequences of *P. sibirica* (*Prunus sibirica* F106 Genome v1.0) were obtained from the Genome Database for Rosaceae (GDR, https://www.rosaceae.org/) (Jung et al., 2019). The hidden markov model (HMM) file of the bHLH domain (PF00010) was downloaded from the Pfam database (http://pfam.xfam.org/) (Mistry et al., 2021) and used for protein screening using HMMER software (version 3.3.2). The online CD-search tool (https://www.ncbi.nlm.nih.gov/Structure/cdd/wrpsb.cgi) (Lu et al., 2020) and Pfam were used to validate the bHLH domains of these protein sequences, and the physical and chemical parameters were calculated using ExPASy (https://www.expasy.org/) (Duvaud et al., 2021). The CELLO program (http://cello.life.nctu.edu.tw/) (Yu et al., 2006) was used to predict the subcellular localization.

### 4.2 Phylogenetic tree and multiple sequence alignment

MEGA software (version 11.0) and Jalview software (version 2.11.2.0) were used to visualize the sequences of the conserved domains in PsbHLHs. The sequence logo for the bHLH domain was created by submitting multiple alignment sequences to WebLogo (http://weblogo.berkeley.edu/logo.cgi) (Crooks et al., 2004).

The full-length bHLHs protein sequences from *P. sibirica* and *A. thaliana* were aligned using ClustalX (version 2.1). Thereafter, a phylogenetic tree was constructed based on the neighbor-joining (NJ) method using MEGA with the following parameter settings: Poisson’s model, pairwise deletion option, and 1000 bootstrap replicates provided for statistical reliability. A phylogenetic tree was constructed using iTOL (https://itol.embl.de/) (Letunic and Bork, 2021). The *A. thaliana* bHLH protein sequences were downloaded from TAIR (https://www.arabidopsis.org/) (Berardini et al., 2015). Subfamily grouping of PsbHLHs was performed according to the AtbHLH classification scheme.

### 4.3 Chromosomal distribution, gene replication, and synteny

Chromosomal locations were identified and plotted using TBtools and the *P. sibirica* genomic database. The nonsynonymous (Ka) and synonymous (Ks) substitution ratios were calculated using the Simple Ka/Ks Calculator in TBtools software to acquire the natural purification selection between target gene pairs. The divergence time was calculated using the formula T = Ks/2r, with Ks being the synonymous substitutions per site, and the r of dicotyledonous plants being 1.5×10^−8^ synonymous substitutions per site per year (Gaut and Doebley, 1997). Finally, sequences with complete bHLH domains were preserved and numbered *PsbHLH1* through *PsbHLH104* to correspond with their locations extending from top to bottom on the chromosome.

The GFF and genome data of *A. thalian*a, *P. persica*, *P. mume*, *P. persica*, and *P. avium* were derived from the GDR. Syntenic analysis maps of *P. sibirica* and other representative plants were constructed using TBtools.

### 4.4 Analysis of gene structure and conserved motif

The structure of *PsbHLHs* was analyzed using TBtools. The conserved motifs of *PsbHLHs* were identified using the online tool MEME (http://meme-suite.org) (Bailey et al., 2015). The sequence of each *PsbHLHs* was retrieved by TBtools according to the genomic full-length DNA sequences of *PsbHLHs*.

### 4.5 Analysis of cis-acting elements and GO annotation

Cis-elements in the promoter (−2000 bp upstream of ATG) were predicted using PlantCARE (https://bioinformatics.psb.ugent.be/webtools/plantcare/html/) (Lescot et al., 2002). Graphs of the structures were drawn using TBtools software.

Eggnog (http://eggnog5.embl.de/) (Huerta-Cepas et al., 2019) was used for GO annotation, and the results were visualized using WeGo (https://wego.genomics.cn/) (Ye et al., 2018).

### 4.6 Prediction of the protein–protein interaction network

All PsbHLH sequences were submitted to STRING (version 11.0, http://string-db.org) (Szklarczyk et al., 2019), and *A. thaliana* was chosen as the reference organism. After the BLAST analysis, the orthologous genes of *A. thaliana* with the highest scores were used to construct a network.

### 4.7 Plant materials and stress treatments

*P. sibirica* clone No. 453 (Appraisal of Improved Cultivar of Forest Trees, S-SV-PS-002-2021, Liaoning Provincial Department of Forest and Grassland) from the National Forest Germplasm Resource Preservation Repository for *Prunus* species at Shenyang Agricultural University (Kazuo, Liaoning, China) was used as the experimental material. The clone No. 453 sprays were placed in an artificial climate box during their peak flowering period and subjected to stress. Samples were collected at 0 h (Control), 15 min, 30 min, 1 h, and 2 h for −4°C low temperature stress. Six different tissue samples (roots, stems, leaves, stamens, pistils, and petals) in a natural environment were collected. All samples were set up with three biological replicates, immediately frozen in liquid nitrogen, and stored at −80°C until required for further analysis.

### 4.8 RNA extraction and qRT-PCR analysis

Total RNA was extracted from the samples using RNA Extraction Kit (Takara, Beijing, China) following the manufacturer’s instructions. The extracted RNA was then reverse transcribed to cDNA using the PrimeScript™ RT Master Mix Perfect Real Time (Takara, Beijing, China), and stored at −80°C. Primer software (version 5.0) was used to design specific primers (**Table S2**), which were synthesized by SYNBIO Technologies (Suzhou, China). The *18S* rRNA gene of *P. sibirica* was used as the internal reference (Kuchipudi et al., 2012). The qRT-PCR was performed using PerfectStart® Green qPCR SuperMix (TransGen Biotech, Beijing, China) in StepOnePlus™ Real-Time PCR System (Applied Biosystems, Foster City, CA, United States) following the manufacturer’s instructions. The qRT-PCR conditions were as follows: 94°C for 30 s, followed by 45 cycles of 94°C for 5 s and 60°C for 15 s, and 72°C for 10 s. The 2^−ΔΔCT^ method was employed to calculate the relative expression levels of *PsbHLHs* (Livak and Schmittgen, 2001).

### 4.9 Construction of *PsbHLH42* overexpression vector and transient transformation in *Populus ussuriensis*

The full-length coding sequence of *PsbHLH42* was cloned into a pRI101-GFP vector. The construct was transformed into *Agrobacterium tumefaciens* GV3101, which was used to transform the tissue culture seedlings of *P. ussuriensis* (Liu et al., 2021).

### 4.10 Determination of physiological parameters

The physiological characteristics of pRI101-GFP empty vector and pRI101-PsbHLH42-GFP seedlings (leaves) were determined after 6 h of 4°C stress treatment. SOD and POD activities were quantified using nitrogen blue tetrazolium (NBT), and guaiacol, respectively. MDA levels were determined using the thiobarbituric acid-reactive substances method. The soluble sugar content (SS) was determined using anthrone colorimetry. Soluble protein content (SP) was determined using the Coomassie Brilliant Blue G-250 staining method. Cell membrane permeability (CMP) was determined using a conductivity meter. The CMP was first determined using a conductivity meter after mixing fresh leaf tissue (0.3 g) with distilled water (15 mL) had been mixed for 4 h. The test tubes were then placed in a boiling water bath for 30 min. After standing for 4 h, measurements were performed again using the same conductivity meter (Wang et al., 2021).

## 5 Conclusion

In our experience, this is the first genome-wide survey of the bHLH gene family in *P. sibirica*. A total of 104 *PsbHLH*s were screened in the *P. sibirica* genome and grouped into 23 subfamilies. *PsbHLHs* were randomly distributed across eight chromosomes. Conserved motifs and exon-intron structures are likely to be the same or similar within the identical subfamily. The duplication of segments is a crucial factor in the expansion of the PsbHLH gene family, and the evolution of *PsbHLHs* is influenced by selective pressure for purification. *PsbHLHs* are primarily involved in biological regulation processes and contain various low-temperature stress- and hormone-related elements. Twenty *PsbHLHs* exhibited diverse expression patterns in response to different low-temperature stresses and different tissues. Overexpression of *PsbHLH42* improved the low-temperature tolerance of *P. ussuriensis*. In summary, these results improve our knowledge of the functional characterization of the PsbHLH gene family and provide genetic resources for cultivating high-quality *P. sibirica* germplasm resources with cold resistance, high yield, and quality.

## Data availability statement

The original contributions presented in the study are included in the article/**Supplementary Files**, further inquiries can be directed to the corresponding author.

## Author contributions

QL: Data curation, Formal Analysis, Funding acquisition, Writing – original draft, Writing – review and editing, Conceptualization. JW: Data curation, Writing – original draft. SW: Data curation, Writing – original draft. JC: Data curation, Writing – original draft. YS: Data curation, Writing – original draft. QL: Investigation, Writing – original draft. XL: Data curation, Writing – original draft. SD: Data curation, Supervision, Writing – review and editing.
