# Supplementary material for: Genome-wide identification, expression analysis, and potential roles under low-temperature stress of bHLH gene family in Prunus sibirica
Source: Front Plant Sci. 2023 Sep 20;14:1267107. doi: 10.3389/fpls.2023.1267107 (PMC10548393; doi:10.3389/fpls.2023.1267107)
Supplement: Supplementary file 1 [file Table_1.docx]

Supplementary Material

# Table S1 The information of conserved motifs in PsbHLHs by MEME tool.

| Motif | Motif Consensus |
| --- | --- |
| 1 | AERRRREKJNERLKALRSLVP |
| 2 | NISKMDKASMLDEAIEYVKELQAQVEELS |
| 3 | RPGLLLKJMEALESLGLQVLHASISTVGG |
| 4 | PPKDYIHVRARRGQATDPHSL |
| 5 | YECERVKEARMHGIQTLVCIPTPCGVLEL |
| 6 | GDVTDSEWFYFYSVSLTQSFAAGHATNNILGRAFCSGGFVW |
| 7 | NFCQENSATFQQRLQFIVQNRPEWWVYSIFWQASKDSNDQVSLSWAGGHF |
| 8 | MIQDVVARVPYGFTSEEAMRMGIIKRWYN |
| 9 | AAEVEVKIVGSEAMIRVQCPK |
| 10 | ASLDVIKEDWGLVHLSKSLFGSDNNRVSK |
| 11 | RRKRRRPRSTKNKEEIESQRMTHIA |
| 12 | MAAMKEMIYRIAAMQPIHIDPESVKPPKRRNVKISKDPQSV |
| 13 | TRQEGGTKEAAPINIGGSSPDSPSDSVGNFTSENTENTRLKKRGRSSNH |
| 14 | ASKRSNKVVNNYQPKFGFNNVERKKVVNR |
| 15 | PQARRPSAALSPEDLTDTEWYYLLCMSFVFPPGEGLPGKALANGQHIWLC |
| 16 | LRPLVRSKEWDYAIFWKJS |
| 17 | MLALSPPLFSTIGWPSEDPJSHDQNYFYRDSFTDQTAESFLHILPSQLPQ |
| 18 | WMKNSADGTEQQVAKLMEEDVGAAMQFLQSKGLCJMPISLAFAIYHTTT |
| 19 | MKLATVNPRLDFNIEGLLSKEILQSR |
| 20 | HHHSSQSTSSIVDHHHSSYNI |

# Table S2 The gene-specific primer sequences.

| gene name | Sense primer | Anti-sense primer |
| --- | --- | --- |
| *PsbHLH5* | 5' GCATCAATCATAGGAGGTG 3' | 5' GGCAATACTACTGGCTTCG 3' |
| *PsbHLH11* | 5' TGGGCAGGCTAATGAACTT 3' | 5' GGATCAGACGCTTGAGACC 3' |
| *PsbHLH19* | 5' CCTCAAATGGGCTCCTACC 3' | 5' GCTTCACGGATTCTGGGTC 3' |
| *PsbHLH24* | 5' GCAAGATGGTGCTAATGGT 3' | 5' TTGTGAAGGTAATGGACGAG 3' |
| *PsbHLH29* | 5' GACATCCAGAGCACCACCAC 3' | 5' TACAATCTTCACCTCCACTTCG 3' |
| *PsbHLH35* | 5' TCACGACCGTCCATCATCT 3' | 5' CTCCTCCGAATCCAAATCC 3' |
| *PsbHLH42* | 5' CAACCATTGACACCCACTCC 3' | 5' CTTCCCTTACCCGAACCTC 3' |
| *PsbHLH40* | 5' GCGAGTTGGTGATGCTGAT 3' | 5' TGGATAGGAAGGAGTGGAGG 3' |
| *PsbHLH39* | 5' TGATGGGACGACTGGTTTAT 3' | 5' GACTGCTTTCCTCCTCTGC 3' |
| *PsbHLH38* | 5' AAGAAGCCCAAACCCAAGT 3' | 5' TTAAGGCGTTCGCTAATCC 3' |
| *PsbHLH46* | 5' GGGTTTGTTGGTGGTGAAG 3' | 5' CAATGTCGGCTCCCTCTT 3' |
| *PsbHLH44* | 5' TCAGGCAGAGGTCAATGTG 3' | 5' TTCAAAGCCAAAGGCAGTC 3' |
| *PsbHLH57* | 5' TTTCACTTCTGCGAATGCC 3' | 5' GAACAACAGAGCGGAGGAC 3' |
| *PsbHLH60* | 5' GGGCTGTGATATTGGGTTTC 3' | 5' TTGAGGCAGACGGGTAGTT 3' |
| *PsbHLH66* | 5' GAGGCCAAGCCACTGATAG 3' | 5' CATTACCGCCATTCCCATT 3' |
| *PsbHLH67* | 5' CTTCAGGGCCACCATCTAC 3' | 5' TGTTACCCGAGCCATCACC 3' |
| *PsbHLH73* | 5' CTCTTTCTGCCATTGTTCC 3' | 5' CAAGCACTTTCACCCTGTC 3' |
| *PsbHLH91* | 5' GCAGCCTTGTAGCAAAGAAATG 3' | 5' GCCTTGAATGAGAATGGGAGTG 3' |
| *PsbHLH89* | 5' GCTGGAGTGGATGAGGATG 3' | 5' TTGGCTTGAAGTGTTTACC 3' |
| *PsbHLH88* | 5' CAAGAACGATAAGGCAACCAT 3' | 5' CACGGGATTCATCAGTAAGC 3' |
| *18S* rRNA | 5' AAACGGCTACCACATCCA 3' | 3'AAACGGCTACCACATCCA 3' |
